# Global and Regional Effects of Bladder Cancer Risk Associated with Pioglitazone Therapy in Patients with Diabetes

**DOI:** 10.1038/s41598-017-16074-1

**Published:** 2017-11-17

**Authors:** Hua Qu, Yi Zheng, Yuren Wang, Rui Zhang, Xiongzhong Ruan, Gangyi Yang, Zhenqi Liu, Hongting Zheng

**Affiliations:** 1 Department of Endocrinology, Xinqiao Hospital, Third Military Medical University, Chongqing, China; 2 John Moorhead Research Laboratory, Royal Free and University College Medical School, University College London, Royal Free Campus, London, United Kingdom; 3 Department of Endocrinology, the Second Affiliated Hospital, Chongqing Medical University, Chongqing, China; 4 Division of Endocrinology and Metabolism, Department of Internal Medicine, University of Virginia Health System, Charlottesville, Virginia USA

## Abstract

It has been debated for several years as to whether the antidiabetic drug pioglitazone increases the risk for bladder cancer. A series of recent large population studies yielded conflicting results. To investigate why the observational studies yielded conflicting results, we conducted stratified analyses to analyze the potential confounders behind these discordant outcomes. A total of 2,764,731 participants from observational (OB) studies and 9,999 from randomized control trials (RCTs) were identified for these analyses. The stratified analysis revealed that the study type, adjustment for age/sex, treatment duration, cumulative dose, agents used in a control group, mean period of follow-up and study population region might contribute to the discordant outcomes. In terms of population regions, pioglitazone increased the risk for bladder cancer could be found in European population, and patients who undergo treatment with pioglitazone for longer durations (>12 months) or are administrated a larger cumulative dose (>28,000 mg) might require more attention, and the long-term effects (≥3.6 years) of pioglitazone needs be monitored more carefully.

## Introduction

Thiazolidinediones (TZDs), agonists of the peroxisome-proliferator-activated receptor (PPAR) γ, reduce blood glucose levels primarily by increasing insulin sensitivity in peripheral tissues, without causing hypoglycemia^1,2^. These agents have been widely used in patients with diabetes mellitus (DM). However, troglitazone was discontinued because it caused hepatotoxicity^3^, and the safety of rosiglitazone was disputed due its effect on the cardiovascular system^4,5^. In contrast, pioglitazone has been shown to prevent the progression to diabetes and major cardiovascular events^6,7^ as well as nonalcoholic steatohepatitis^6^, thereby indicating a broader prospect for its clinical applications. However, globally, safety concerns pertaining to the potential effect of pioglitazone in increasing bladder cancer risk have been raised and debated for many years^8–23^. A series of large clinical studies^24–26^, such as the Kaiser Permanente Northern California (KPNC) study, United Kingdom Clinical Practice Research Datalink (UKCPRD) research, and Four European Countries Datasets (FECD) research, obtained discordant results, which led to more debate regarding this issue. Although this observation is still debated, the U.S. Food and Drug Administration (FDA) did warn about this risk on December 12, 2016 (http://www.fda.gov/Drugs/DrugSafety/ucm519616.htm).

We conducted stratified analyses to investigate why the observational studies yield conflicting results and to analyze potential confounders that caused inconsistent results in the previous studies, as well as to determine optimal future study designs. In addition, as pioglitazone use may be related to other malignancies as well^24,27^, risk for other cancers types were also evaluated in the study.

## Methods

### Literature Search

Embase, PubMed, Web of Science, Cochrane Central Register of Controlled Trials (CENTRAL), and ClinicalTrials.gov were searched from inception through Jan 5, 2017 without language restriction. Two independent reviewers (Y.W. and R.Z.) searched and selected studies separately. Disagreements were resolved by discussion between the reviewers, and if necessary, consultation with other authors included in this study. Our search strategy included the following terms pertinent to pioglitazone: peroxisome proliferator activated receptor agonist/activator, PPAR, thiazolidinediones, TZDs, pioglitazone, Actos; and terms pertinent to cancer: cancer, tumor, carcinoma, neoplasm, malignancy (eAppendix 1 in the supplement). References of relevant studies were manually screened for eligible sources of data.

### Inclusion and Exclusion Criteria

All human studies that evaluated patients with DM, reported pioglitazone therapy, and provided cancer outcomes were included. Observational (OB) studies and randomized controlled trials (RCTs) that provided either relative risk estimates such as risk ratios (RR), hazard ratios (HR) or odds ratios (OR) and 95% confidence intervals (CI) for cancers or raw data were eligible. Studies with the greatest number of patients and the latest publications were selected when overlapping subjects were included in more than one study. Trials reporting serious adverse events or adverse events related to cancer following pioglitazone exposure were also included.

We excluded duplicate reports and abstracts from meeting proceedings. Studies were also excluded if they were animal research, reviews, comments or replies.

### Data Extraction

Three reviewers (Y.W., H.Q. and R.Z.) independently extracted data from the primary texts and supplementary appendixes of all trials. Disagreements among the three reviewers were resolved by discussion, and if necessary, consultation with the other two reviewers (Y.Z. and H.Z.). The following data were collected for OB studies: authors, year of publication, age and sex, sample size, number of cancer events in both groups, RR, HR, OR with 95% CI, study type, adjustment factors, dose/duration response gradient, types of medications used in exposed and control groups, mean period of follow-up, target disease, and population region. For RCTs, data included trial registry number, number of study sites, study phase, number of cancer events in both groups, types of medications used in interventional and control groups, duration of follow-up, target disease, and population region.

For relative risk estimates of each study, we selected the most adjusted value (that is, the multivariable association measure with the highest number of covariates, to reduce the biases as much as possible) and corresponding 95% CI, in addition to raw events data. Unadjusted estimates were selected if the outcome was not adjusted for any variable.

### Quality Assessment

Three reviewers (Y.W., R.Z. and H.Q.) independently assessed the quality of all studies. The Newcastle-Ottawa quality assessment scale^28^ was used to assess the risk of bias of cohort studies and case-control studies. The highest-quality score was 9 (maximum), and studies with scores ≥7 were considered as having a low risk of bias, scores of 4–6 as having a moderate risk of bias, and scores <4 as having a high risk of bias. The item “was follow-up long enough for outcomes to occur” for cohort studies was removed owing to the adequate duration of follow-up is uncertain and was analyzed as a result of this study. The Cochrane Collaboration’s tool^29^ was used to assess the risk of bias of RCTs. The judgmental items were “random sequence generation”, “allocation concealment”, “blinding of participants and personnel”, “blinded assessment of bladder cancer events”, “incomplete outcome data”, and “selective reporting”.

### Definitions

The primary outcomes were included to examine the association between pioglitazone use and bladder cancer risk and whether this association varied based on the study design (i.e., study type, adjusted factors, intervention measures, comparator agents, follow-up duration, and study population)^30^. The secondary outcome was defined as the relationship between pioglitazone use and other cancer risks.

### Statistical Analysis

As the incidence rates of cancers involved in our study are relatively rare (<5%) in overall and subgroup analyses, the distinctions among the RR, HR, and OR can be ignored^31^. We pooled relative risk estimates and reported pooled OR with corresponding 95% CI using random effects models according to the methodology proposed by DerSimonian and Laird^32^, with weights calculated by the inverse variance method in cases of heterogeneity. Otherwise, fixed-effects models were used. Subgroup analyses were conducted to explore whether the association between pioglitazone use and bladder cancer risk were varied by study design.

The MOOSE guidelines^33^ for meta-analysis were followed, and PRISMA criteria^34^ were performed for reporting our meta-analysis. Publication bias was evaluated visually by funnel plots and quantified by the Egger’s test and the Begg’s test^35,36^. Heterogeneity across trials was assessed by the*I* ^2^ statistic, with values greater than 50% indicating significant statistical heterogeneity^37^. If significant statistical heterogeneity was detected, a sensitivity analysis was conducted using the “leave one out” approach^38^ to identify the source of heterogeneity. This “leave one out” approach was also used for sensitivity analysis to detect the influence of a single study on the overall bladder cancer risk. In addition, sensitivity analyses were also conducted by study quality analysis, which was restricted to the highest-quality studies with Newcastle-Ottawa scale scores of 8–9, and to test the robustness of overall bladder cancer risk and subgroup analysis.

Results with 2-sided *P*-values less than 0.05 were considered statistically significant. All statistical analyses were performed using Stata Statistical Software: version 12.0 (STATA Corp, College Station, TX).

## Results

**Figure 1:**
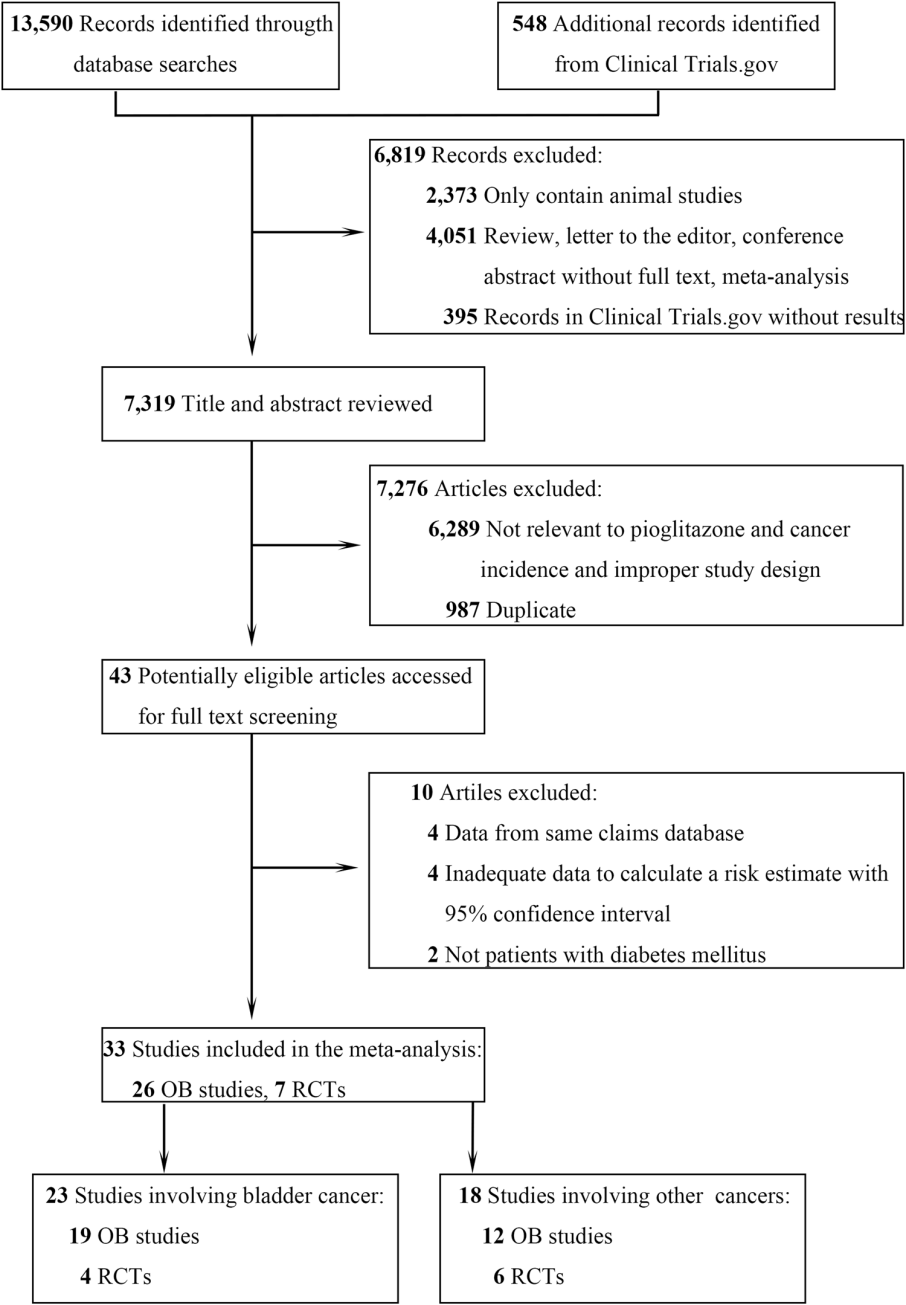
Flowchart of the Literature Search and Study Selection Abbreviations: OB, observational studies. RCTs, randomized controlled trials.

Figure 1 outlines the procedure used for the literature search. For analysis of the primary outcomes (see Definitions in Methods), 19 OB studies^9,12–22,24–27,39–41^ and 4 RCTs (including clinical trials NCT00494312 and NCT00736099)^10,42^ published between 1986 and 2016 were identified. From the OB studies, 2,764,731 participants were included. Of these participants, 343,176 (12.4%) were exposed to pioglitazone, and 13,264 (0.5%) developed bladder cancer. The mean period of follow-up ranged from 2.1 to 7.9 years. In the RCTs, 9,999 participants were included. Of these participants, 4,515 (45.2%) were exposed to pioglitazone, and 24 (0.2%) developed bladder cancer. The mean period of follow-up ranged from 72 weeks to 48 months (eTable 1 and eReferences in the Supplement). For analysis of the secondary outcome (see Definitions in Methods), 12 OB studies^9,13,17,24,27,39,43–48^ and 6 RCTs (including clinical trials NCT00736099, NCT00676338, NCT00879970, and NCT00637273)^10,42^ were identified, and 17 other site-specific cancers were assessed.

### Part 1: Analysis with observational studies

#### Pioglitazone and overall bladder cancer risk at the global level

**Figure 2:**
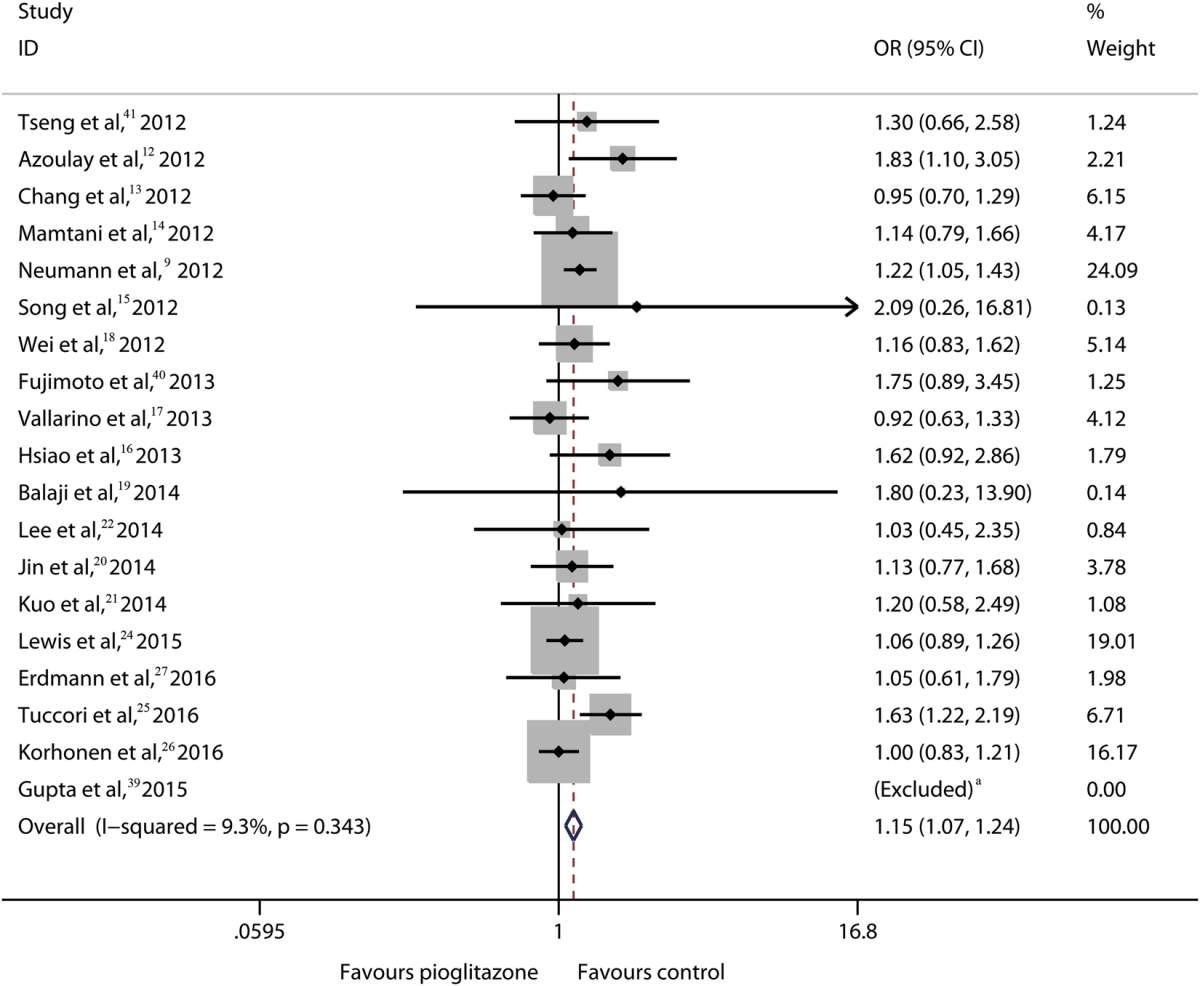
Bladder Cancer Risks Related to Pioglitazone Use Versus Control for Patients with DM in Global from OB Studies Abbreviations: DM, diabetes mellitus. ^a^Given that both the exposed and control groups did not report bladder cancer incidence, the OR was not estimable.

First, we conducted a pooled analysis to determine whether pioglitazone use was associated with the bladder cancer risk at the global level. To date, a majority of the studies related to this topic are OB studies. Only 4 RCTs were identified; thus, at first the OB studies were analyzed. The results showed that the risk for bladder cancer risk increased by 15% (OR, 1.15; 95% CI, 1.07–1.24; *P* < 0.001) **(**Fig. 2 **)**, indicating that pioglitazone use was associated with overall bladder cancer risk based on the existing evidence.

#### Study type and pioglitazone related bladder cancer risk at the global level

**Figure 3:**
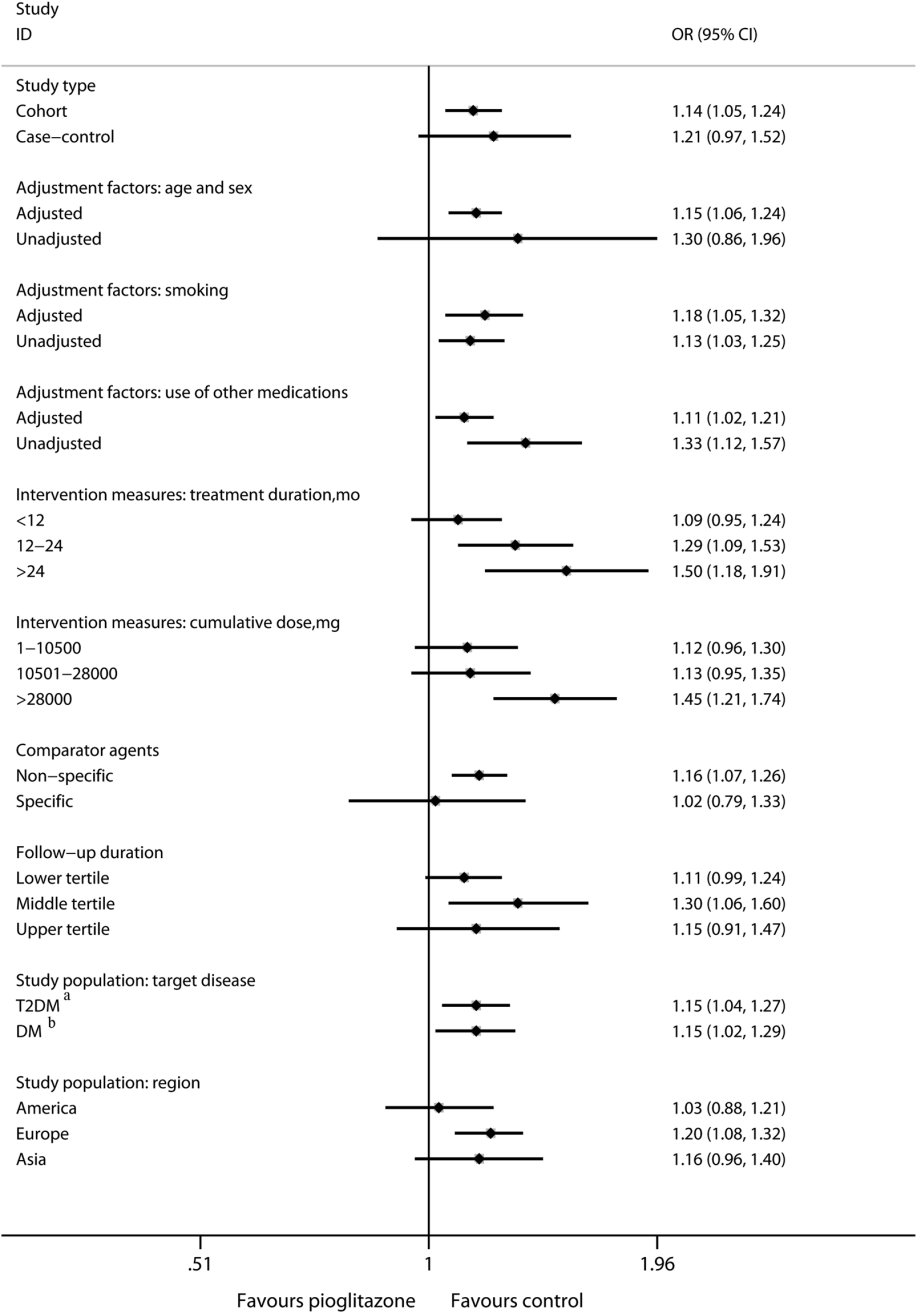
Subgroup Analyses of Bladder Cancer Risk Related to Pioglitazone Use Versus Control for Patients with DM in Global from OB Studie. Abbreviations: T2DM, type 2 diabetes mellitus; T1DM, type 1 diabetes mellitus. ^a^With T1DM excluded. ^b^With T1DM included.

Next, to explore the reason behind the inconsistent outcomes observed in previous individual studies, the potential confounders for study outcome, which are generally derived from study design (study type, adjustment factors, intervention measures, comparator agents, follow-up duration, and study population)^30^, were analyzed. In terms of the study type, cohort studies showed a positive result with regard to the relationship between pioglitazone and bladder cancer risk (OR, 1.14; 95% CI, 1.05–1.24; *P* = 0.001), whereas case control studies showed negative results (OR, 1.21; 95% CI, 0.97–1.52; *P* = 0.10) **(**Fig. 3 **)**, suggesting that study type might be a confounder for the global outcomes.

#### Adjustment factors and pioglitazone related bladder cancer risk at the global level

With regard to adjustment factors, the main cancer risk factors that are commonly adjusted in medication related cancer risk studies include age/sex, smoking, unhealthy diet and lack of physical activity, and the use of other medications^49^. Although the risk factor “unhealthy diet and lack of physical activity” could not be analyzed (refer to the limitations list in the Discussion section for the reasons), the remaining 3 factors were evaluated. The adjustment for both smoking and use of other medications did not influence the outcomes (smoking: adjusted, OR, 1.18; 95% CI, 1.05–1.32; *P* = 0.006 vs. non-adjusted, OR, 1.13; 95% CI, 1.03–1.25; *P* = 0.01; use of other medications: adjusted, OR, 1.11; 95% CI, 1.02–1.21; *P* = 0.02 vs. non-adjusted, OR, 1.33; 95% CI, 1.12–1.57; *P* = 0.001), whereas adjustment for age/sex significantly affected the results (adjusted: OR, 1.15; 95% CI, 1.06–1.24; *P* = 0.001 vs. non-adjusted: OR, 1.30; 95% CI, 0.86–1.96; *P* = 0.22) **(**Fig. 3 **)**. These results indicated that adjustment for age/sex might also be a confounder that affected previous outcomes, and the results of the studies adjusted for age/sex seem more credible.

#### Intervention measures and pioglitazone related bladder cancer risk at the global level

With regard to intervention measures, administration routes as well as the treatment duration and cumulative dose were commonly considered^50–52^. However, pioglitazone is only orally administrated, only the duration and cumulative dose were assessed in this study. Of the included OB trials, in 13, the duration or cumulative dose-response relation was evaluated, and in 8, the unified categories were used. These 8 trials were analyzed. Increased risks of bladder cancer were identified in longer duration and larger cumulative dose subgroups (duration: 12–24 months, OR, 1.29; 95% CI, 1.09–1.53; *P* = 0.003; >24 months, OR, 1.50; 95% CI, 1.18–1.91; *P* = 0.001; cumulative dose: >28,000 mg, OR, 1.45; 95% CI, 1.21–1.74; *P* < 0.001), while the shorter duration and smaller cumulative dose subgroups did not increased risks of bladder cancer (duration: <12 months, OR, 1.09; 95% CI, 0.95–1.24; *P* = 0.21; cumulative dose: 1–10,500 mg, OR, 1.12; 95% CI, 0.96–1.30; *P* = 0.17; 10,501–28,000 mg, OR, 1.13; 95% CI, 0.95–1.35; *P* = 0.16) **(**Fig. 3 **)**. These results indicated that the treatment duration and cumulative dose might also be confounders, and that long durations and large cumulative doses of pioglitazone use should be carefully monitored.

#### Comparator agents and pioglitazone-related bladder cancer risk at the global level

The medication used in the control group can also be a potential confounder^53^. Of the included OB trials, non-specific agents were used in the control groups of 17 studies, and specific agents (insulin and rosiglitazone) were reported in 2. A positive relationship was identified in the non-specific comparator subgroup (OR, 1.16; 95% CI, 1.07–1.26; *P* < 0.001), whereas the result was negative in the specific comparator subgroup (OR, 1.02; 95% CI, 0.79–1.33; *P* = 0.86) **(**Fig. 3 **)**, indicating that comparator agents might influence the outcomes. Moreover, insulin and rosiglitazone have been reported to potentially influence bladder cancer risks^54,55^, this likely leads to the incorrect estimation of the association between pioglitazone use and bladder cancer risk. Thus, the results of studies that used non-specific agents as the control group might be more reasonable.

#### Follow-up duration and pioglitazone related bladder cancer risk at the global level

The follow-up duration mainly refers to the mean period of follow-up, as the maximum period cannot describe the maturity of the study data and the quality of follow-up^56^. This factor was analyzed using the tertiles method (the lower and upper cut-off points were 3.6 and 4.8 years, respectively) in our study. The middle tertile exhibited a positive association between pioglitazone and bladder cancer risk (OR, 1.30; 95% CI, 1.06–1.60; *P* = 0.01), whereas the lower (OR, 1.11; 95% CI, 0.99–1.24; *P* = 0.08) and upper tertiles (OR, 1.15; 95% CI, 0.91–1.47; *P* = 0.25) did not show any association **(**Fig. 3 **)**, indicating that the follow-up duration might influence the outcomes. However, the pooled result of the upper tertile was inexplicable at the global level.

#### Study population and pioglitazone related bladder cancer risk at the global level

The study population commonly includes the target disease and the population region^57^. In terms of target disease, the relationship between pioglitazone use and bladder cancer risk was positive both in the T2DM (with T1DM excluded) and DM (with T1DM included) subgroups (T2DM: OR, 1.15; 95% CI, 1.04–1.27; *P* = 0.006 vs. DM: OR, 1.15; 95% CI, 1.02–1.29; *P* = 0.02) **(**Fig. 3 **)**. However, for the population region, the European region exhibited an increased risk of bladder cancer after pioglitazone exposure (OR, 1.20; 95% CI, 1.08–1.32; *P* < 0.001), whereas the results in both American (OR, 1.03; 95% CI, 0.88–1.21; *P* = 0.68) and Asian (OR, 1.16; 95% CI, 0.96–1.40; *P* = 0.12) regions were negative **(**Fig. 3 **)**, indicating that the population region might be a confounder at the global level.

#### Study design and pioglitazone related bladder cancer risk in different population regions

**Figure 4:**
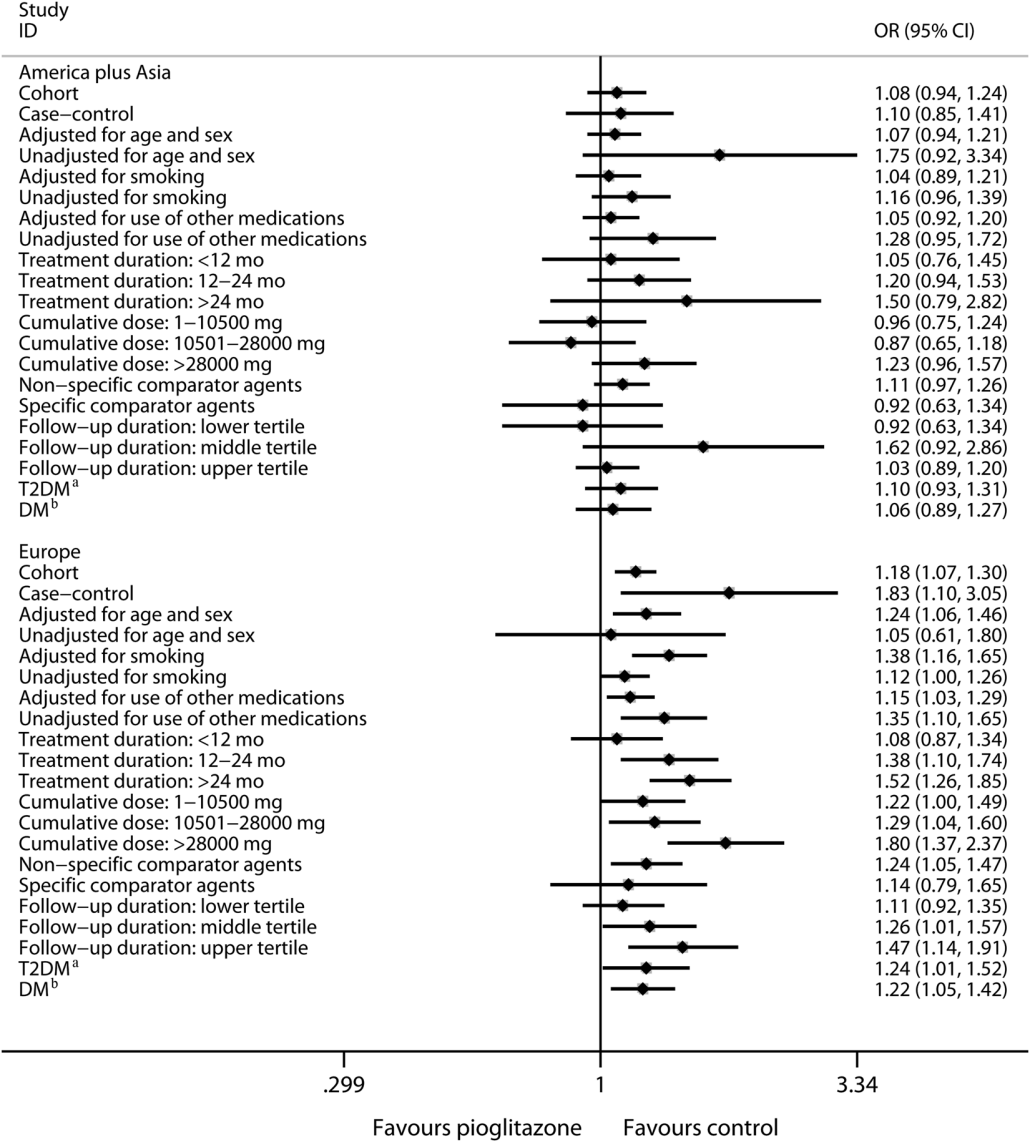
Subgroup Analyses of Bladder Cancer Risk Related to Pioglitazone Use Versus Control for Patients with DM in Europe, and America plus Asia, Respectively from OB Studies. ^a^With T1DM excluded. ^b^With T1DM included.

The population region is a confounder that influences outcomes objectively^58–60^. Therefore, we re-analyzed the potential confounders in different population regions. For American and Asian regions, the results were negative (*P* > 0.05), further confirming the negative pooled results **(**Fig. 4, Table 1 **)**. In the European region, the results were positive (*P* < 0.05) except for the subgroups unadjusted for age/sex (OR, 1.05; 95% CI, 0.61–1.80; *P* = 0.86), moking (OR, 1.12; 95% CI, 1.00–1.26; *P* = 0.052), the shortest treatment duration (OR, 1.08; 95% CI, 0.87–1.34; *P* = 0.49) and follow-up period (OR, 1.11; 95% CI, 0.92–1.35; *P* = 0.28), and specific agent (rosiglitazone) used in the control group (OR, 1.14; 95% CI, 0.79–1.65; *P* = 0.49). As mentioned above, the results of studies adjusted for age/sex and smoking, with longer treatment durations, and non-specific agents used in the control group were considered more credible; therefore, the positive results achieved in these subgroups in both Europe and globally further confirmed the positive pooled European and global results (Fig. 4, Table 1). Interestingly, the subgroup results of the longest follow-up were positive in the European populations (OR, 1.47; 95% CI, 1.14–1.91; *P* = 0.003) but negative in the American and Asian populations (OR, 1.03; 95% CI, 0.89–1.20; *P* = 0.68). This finding might explain the inexplicable negative result of the longest follow-up subgroup at global level (Fig. 4, Table 1). Moreover, when the bladder cancer risk related to pioglitazone was considered based on different population regions, the study type no longer influenced the outcomes (Fig. 4, Table 1). Taken together, these results indicated that a positive relationship between pioglitazone and bladder cancer risk may be present in the European population. Thus, it is important to pay more attention to European patients, especially those in whom the treatment duration is long; the long-term effects of pioglitazone need to be monitored closely in these cases. In addition, future related studies should adjust for age/sex and smoking, and adopt non-specific agents in the control group.

**Table 1:** Bladder Cancer Risk Related to Pioglitazone Use in Patients with DM Compared Globally and Regionally from OB Studies.

	Global			Sub-regional					
	Outcome^a^	OR (95% CI)	*P*-Value	American plus Asian Regions			European Region		
				Outcome^a^	OR (95% CI)	*P*-Value	Outcome^a^	OR (95% CI)	*P*-Value
**Study type**									
Cohort	+	1.14 (1.05, 1.24)	0.001	—	1.08 (0.94, 1.24)	0.26	+	1.18 (1.07, 1.30)	0.001
Case-control	—	1.21 (0.97, 1.52)	0.10	—	1.10 (0.85, 1.41)	0.47	+	1.83 (1.10, 3.05)	0.02
**Adjustment factors**									
**Age and sex**									
Adjusted	+	1.15 (1.06, 1.24)	0.001	—	1.07 (0.94, 1.21)	0.30	+	1.24 (1.06, 1.46)	0.009
Unadjusted	—	1.30 (0.86, 1.96)	0.22	—	1.75 (0.92, 3.34)	0.09	—	1.05 (0.61, 1.80)	0.86
**Smoking**									
Adjusted	+	1.18 (1.05, 1.32)	0.006	—	1.04 (0.89, 1.21)	0.64	+	1.38 (1.16, 1.65)	< 0.001
Unadjusted	+	1.13 (1.03, 1.25)	0.01	—	1.16 (0.96, 1.39)	0.13	—	1.12 (1.00, 1.26)	0.052
Use of other medications									
Adjusted	+	1.11 (1.02, 1.21)	0.02	—	1.05 (0.92, 1.20)	0.45	+	1.15 (1.03, 1.29)	0.01
Unadjusted	+	1.33 (1.12, 1.57)	0.001	—	1.28 (0.95, 1.72)	0.11	+	1.35 (1.10, 1.65)	0.004
**Intervention measures**									
**Treatment duration, mo**									
< 12	—	1.09 (0.95, 1.24)	0.21	—	1.05 (0.76, 1.45)	0.78	—	1.08 (0.87, 1.34)	0.49
12–24	+	1.29 (1.09, 1.53)	0.003	—	1.20 (0.94, 1.53)	0.14	+	1.38 (1.10, 1.74)	0.006
> 24	+	1.50 (1.18, 1.91)	0.001	—	1.50 (0.79, 2.82)	0.21	+	1.52 (1.26, 1.85)	< 0.001
**Cumulative dose, mg**									
1–10500	—	1.12 (0.96, 1.30)	0.17	—	0.96 (0.75, 1.24)	0.78	+	1.22 (1.00, 1.49)	0.048
10501–28000	—	1.13 (0.95, 1.35)	0.16	—	0.87 (0.65, 1.18)	0.37	+	1.29 (1.04, 1.60)	0.02
> 28000	+	1.45 (1.21, 1.74)	< 0.001	—	1.23 (0.96, 1.57)	0.10	+	1.80 (1.37, 2.37)	< 0.001
**Comparator agents**									
**Agents use in control group**									
Specific	+	1.16 (1.07, 1.26)	< 0.001	—	1.11 (0.97, 1.26)	0.12	+	1.24 (1.05, 1.47)	0.01
Non-specific	—	1.02 (0.79, 1.33)	0.86	—	0.92 (0.63, 1.34)	0.66	—	1.14 (0.79, 1.65)	0.49
**Follow-up Duration**									
**Mean period of follow-up, y**									
Lower tertile, < 3.6	—	1.11 (0.99, 1.24)	0.08	—	0.92 (0.63, 1.34)	0.66	—	1.11 (0.92, 1.35)	0.28
Middle tertile, 3.6–4.8	+	1.30 (1.06, 1.60)	0.013	—	1.62 (0.92, 2.86)	0.10	+	1.26 (1.01, 1.57)	0.04
Upper tertile, ≥ 4.8	—	1.15 (0.91, 1.47)	0.25	—	1.03 (0.89, 1.20)	0.68	+	1.47 (1.14, 1.91)	0.003
**Study population**									
**Target disease**									
T2DM^b^	+	1.15 (1.04, 1.27)	0.006	—	1.10 (0.93, 1.31)	0.24	+	1.24 (1.05, 1.47)	0.01
DM^c^	+	1.15 (1.02, 1.29)	0.02	—	1.06 (0.89, 1.27)	0.48	+	1.14 (0.79, 1.65)	0.49
**Study population region**									
America	—	1.03 (0.88, 1.21)	0.68	NA	NA	NA	NA	NA	NA
Europe	+1	1.20 (1.08, 1.32)	< 0.001	NA	NA	NA	NA	NA	NA
Asia	—	1.16 (0.96, 1.40)	0.12	NA	NA	NA	NA	NA	NA

Abbreviations: DM, diabetes mellitus; OB, observational studies; T2DM, type 2 diabetes; T1DM, type 1 diabetes; NA, not available.

^a^The outcome includes statistical increased risk of bladder cancer ( + ) and non-association (-) related to pioglitazone use.

^b^With T1DM excluded.

^c^With T1DM included.

#### Pioglitazone and other cancer risks

**Figure 5:**
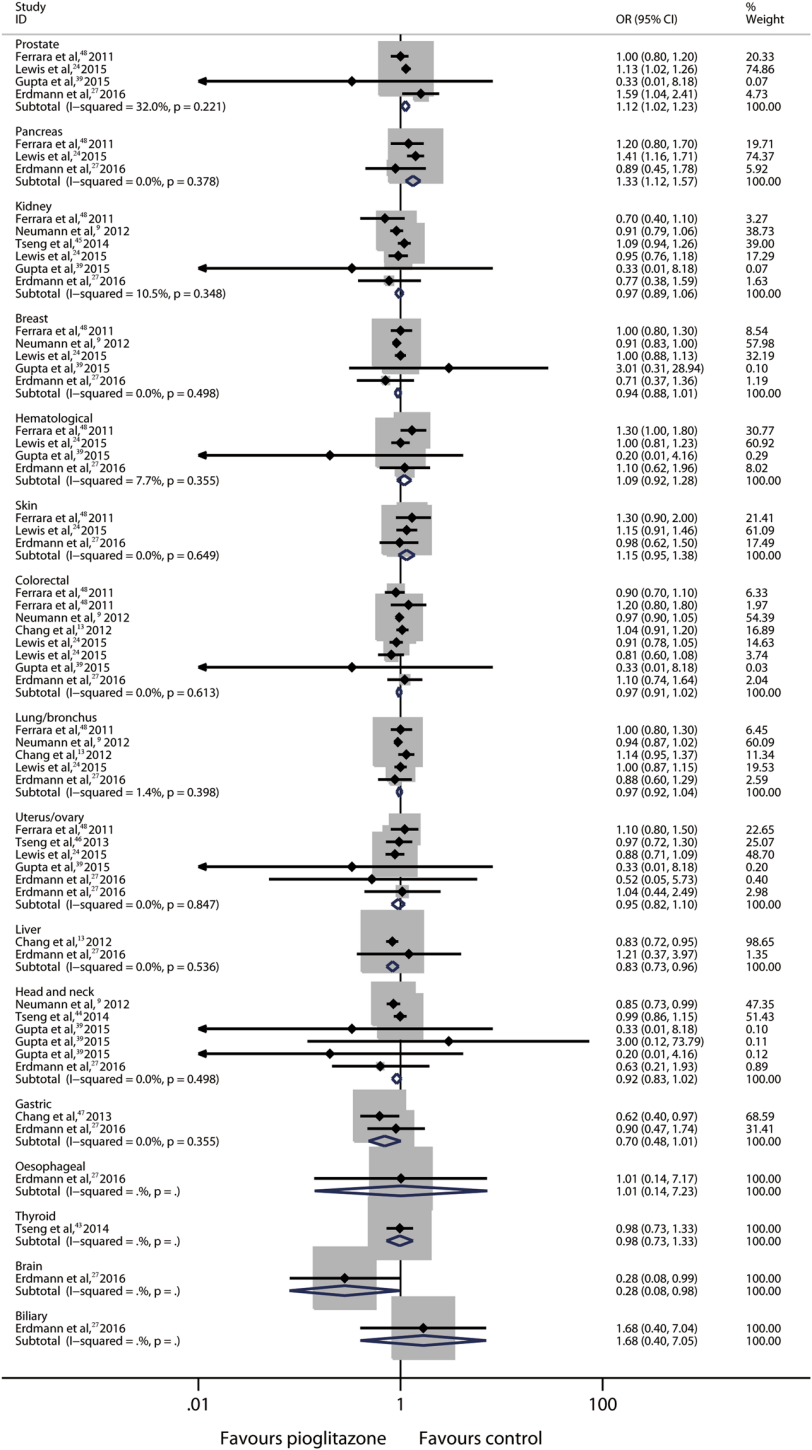
Other Cancer Risks in Patients with DM Receiving Pioglitazone Versus Control from OB Studies^a^. ^a^With Vallarino *et al*.^17^ excluded.

In addition to bladder cancer, pioglitazone exposure has also been reported to be associated with prostate and pancreatic cancer risks^24,27^. To examine the relationship between pioglitazone and the other cancers types, 16 site-specific cancers assessed in 12 related OB studies were analyzed. However, one-third of the results of the initial analysis yielded significant heterogeneities (*I* ^2^ > 50%, esophagus, thyroid, brain and biliary cancers were only reported by one study each, and it was not possible to test heterogeneity, eFigure 1 in the Supplement). Therefore, sensitivity analyses were conducted using the “leave one out” approach^38^, and one study (Vallarino *et al*.^17^) was identified as a common contributor for these heterogeneities (*I* ^2^ decreased to ≤32% with Vallarino *et al*.^17^ excluded) **(**Fig. 5 **)**. The re-summary results showed that pioglitazone use was associated with increased risks of prostate and pancreatic cancer (prostate: OR, 1.12; 95% CI, 1.02–1.23; *P* = 0.02; pancreatic: OR, 1.33; 95% CI, 1.12–1.57; *P* = 0.001), and decreased risks of liver and brain cancer (liver: OR, 0.83; 95% CI, 0.73–0.96; *P* = 0.01; brain: OR, 0.28; 95% CI, 0.08–0.98; *P* = 0.047, with only one report) **(**Fig. 5 **)**. These results indicated that pioglitazone use might also be related to other cancer risks.

### Part 2: Reanalysis using OB studies and RCTs

Heretofore, only 4 RCTs related to pioglitazone and bladder cancer risk and a positive pooled result of RCTs have been reported^61^. Therefore, we combined all the OB studies with the 4 RCTs and reanalyzed the results. Given that the 4 RCTs were not involved in the adjustment factors and intervention measures analyses, we included comparator agents, follow-up duration, and study population in the subgroup analyses. As for other cancers, 17 site-specific cancers were analyzed. All the re-analytical results with OB studies plus RCTs were similar to those obtained from the OB studies (eFigure 2–5 in the Supplement).

#### Sensitivity Analysis

For overall bladder cancer risk, sensitivity analyses were performed both using “study quality” analysis (eTable 2 in the supplement) and using the “leave one out” approach, and none of the results showed significant changes. For the subgroup analyses of bladder cancer risk, “study quality” sensitivity analysis was conducted, and the results remained similar. With regard to risks for other cancer types, a sensitivity analysis was conducted using the “leave one out” approach, the results of which are presented in the “Pioglitazone and other cancer risks” section of the Results.

#### Publication Bias Analysis

No significant bias was detected by Egger’s test (*P* = 0.22) or Begg’s test (*P* = 0.57 [continuity corrected]) (eFigure 6, 7 in the supplement).

## Discussion

Global safety concerns about the association of pioglitazone use with bladder cancer risk have been present and debated since the PROspective pioglitAzone Clinical Trial in macroVascular Events (PROactive) study was conducted, more than a decade ago^10^. Recently, the discordant results obtained from several large-population studies further increased the uncertainty^24–26^. Here, we identified certain regional differences that might be extremely important factors that contribute to the inconsistent outcomes of the previous studies.

Our analysis suggested that pioglitazone use was associated with bladder cancer risk at the global level (15% increase), which was consistent with the results of most of the previous meta-analyses (17% to 23% increase)0^51,52,61–64^. In addition, the negative results were achieved from the subgroups unadjusted for age/sex, short treatment durations and small cumulative doses, and the specific agents (insulin and rosiglitazone) used as comparators during the stratified analysis with potential confounders. These also strengthen the positive relationship, as adjusted results are more credible, and diabetic patients require long-term treatment^65^, and hence insulin and rosiglitazone may influence bladder cancer risk^54,55^.

Most importantly, our results indicated for the first time that the association of pioglitazone use and bladder cancer risk exhibits a significant regional variation. While the pooled and stratified results were negative in the American and Asian regions, results from studies conducted in the European region showed a striking positivity from the pooled and most of the stratified analyses. Although the subgroups unadjusted for age/sex and smoking, short treatment duration and follow-up period, and rosiglitazone used in the control group showed negative results, based on the reasons mentioned above, the results further confirmed a positive relationship in European populations. This regional difference might explain the discordant results of several previous studies, such as the KPNC and UKCPRD^24,25^. Similarly, the risk factors for stroke, disabling sequelae from bacterial meningitis, and obesity were also reported to have regional differences^58–60^. Interestingly, when the relationship was considered in terms of different population regions, the study type no longer influenced the outcomes, and the inexplicable results of follow-up duration analysis at the global level could be interpreted reasonably. Based on these findings, we suggest that the bladder cancer risk related to pioglitazone should be considered according to the different population regions, and the European population require more attention. Notably, although we identified the regional differences across continents, the possibility of variation among different countries and regions may exist. Whether the regional differences that we observed in this study are associated with race and ethnicity remain unclear, as only the KPNC study^24^ described the racial composition of its study population. Further studies are required to clarify these issues.

Moreover, when performing future studies, the authors need to be aware of the long-term effects of prolonged pioglitazone use and should adjust for age/sex and smoking and adopt non-specific agents in the control group. Although our results indicated that the study design did not influence the outcomes of the American and Asian populations, it still had an effect on the European population. In the European population, positive results were achieved with long treatment durations (>12 months) and follow-up periods (≥3.6 years); therefore, we should pay more attention to this population. In addition, adjustment for age/sex and smoking, and the specific comparator agent (rosiglitazone) influenced the outcomes; therefore, these factors should also be noticed in future related studies.

Our results indicated that pioglitazone use might also be related to increased risk for other cancer types, such as prostate and pancreatic cancer, which is consistent with the observations of the KPNC study^24^, and decreased risks for liver and brain cancer. Additionally, one study recently suggested that pioglitazone might influence cancer progression^66^. However, studies on these other cancers are very limited and more studies are needed to answer several key questions in this field: Does pioglitazone influence the risk and/or progression of other cancers? Does the association between pioglitazone use and risk for other cancer types show regional differences? If so, what is the mechanism behind the role of pioglitazone in tumorigenesis?

Our study has important implications in future clinical practice and trials in that (1) more attention regarding bladder cancer risk related to pioglitazone should be given to European, especially those with long treatment durations and a large cumulative dose; (2) studies should adjust for age/sex and smoking, and adopt non-specific agents in the control group; and (3) the risks of other site-specific cancers, such as prostate, pancreatic, liver and brain cancer, need to be addressed.

These are several limitations to our study. First, among the main cancer risk factors, the factor “unhealthy diet and lack of physical activity” was not analyzed because it was reported by only one study^12^ in the form of alcohol intake. Second, some factors that were adjusted for in previous individual trials were not analyzed for the following reasons. Only one^18^ and two trials^22,24^ have adjusted for body mass index (BMI) and income, respectively; therefore, the data are inadequate for meaningful analyses. The comorbidities selected for adjustment different from trial to trial, and the diabetes duration and date of cohort entry were defined inconsistently among the trials^9,12–22,24–27,39–41^; thus, these 3 factors were also unsuitable for analysis. Only 3 trials adjusted for hemoglobin A1c concentration (HbA1c)^12,24,25^. Although the analyzed results were accordant with our conclusions, more data might be required for further analysis. The results obtained from analysis at the global level showed that adjustment for HbA1c affected the outcome (adjusted: OR, 1.40; 95% CI, 0.98–2.02; *P* = 0.07 vs. non-adjusted: OR, 1.12; 95% CI, 1.03–1.23; *P* = 0.01, efigure 8 in the supplement). However, only 3 adjusted trials were included, of which 2^12^^,^^25^ were conducted in Europe and 1^24^ was conducted in America; thus, this regional difference might contribute to the negative pooled results of the adjusted trials at the global level. When the association was analyzed by region, the results of both the HbA1c-adjusted and unadjusted groups were positive in the European regions (adjusted: OR, 1.68; 95% CI, 1.30–2.16; *P* < 0.001 vs. non-adjusted: OR, 1.13; 95% CI, 1.01–1.25; *P* = 0.03) but negative in the American and Asian regions (adjusted: OR, 1.06; 95% CI, 0.89–1.26; *P* = 0.51 vs. non-adjusted: OR, 1.11; 95% CI, 0.94–1.31; *P* = 0.23) (efigure 9 in the supplement), which further confirmed our conclusions. Third, the variation of diagnostic criteria for bladder cancer and other cancers among these studies could not be assessed in our study. Finally, most of the included studies were observational in design and patient-level data for each of the studies could not be obtained due to authorization limit. However, most of these studies have adjusted for major potential confounders, such as age/sex, smoking, and comorbidities, for bias control.

## Conclusions

In summary, the risk of bladder cancer with pioglitazone use might needs to be considered in the European population, and patients with a longer treatment duration (>12 months) or a larger cumulative dose (>28,000 mg) might should be followed up more carefully. The long-term effects (≥3.6 years) of pioglitazone might also need to be noted. In addition, future related studies should adjust for age/sex and smoking and adopt non-specific agents in the control group. Moreover, pioglitazone use may relate to risks for other cancer types.

### Access to research materials

Information about how the data can be accessed is available from the corresponding author.

## Electronic supplementary material

Supplementary material
