# Supplementary material for: Global and Regional Effects of Bladder Cancer Risk Associated with Pioglitazone Therapy in Patients with Diabetes
Source: Sci Rep. 2017 Nov 17;7:15804. doi: 10.1038/s41598-017-16074-1 (PMC5694000; doi:10.1038/s41598-017-16074-1)
Supplement: Supplementary file 1 — Supplementary material [file 41598_2017_16074_MOESM1_ESM.pdf]

# Global and Regional Effects of Bladder Cancer Risk Associated with Pioglitazone Therapy in Patients with Diabetes

## A Systematic Review and Meta-Analysis

Hua Qu[†1], M.D.; Yi Zheng[†1], Ph.D.; Yuren Wang[†1], M.D.; Rui Zhang[1], M.D.; Xiongzhong Ruan[2], Ph.D.; Gangyi Yang[3], M.D.; Zhenqi Liu[4], M.D.; Hongting Zheng[*1], M.D.

**Author Affiliations:**

1 Department of Endocrinology, Xinqiao Hospital, Third Military Medical University, Chongqing, China (Qu, Y. Zheng, Wang, Zhang, and HT. Zheng);

2 John Moorhead Research Laboratory, Royal Free and University College Medical School, University College London, Royal Free Campus, London, United Kingdom (Ruan);

3 Department of Endocrinology, the Second Affiliated Hospital, Chongqing Medical University, Chongqing, China (Yang);

4 Division of Endocrinology and Metabolism, Department of Internal Medicine, University of Virginia Health System, Charlottesville, Virginia, USA (Liu).

**Corresponding Author:** Hongting Zheng, M.D., Department of Endocrinology, Xinqiao Hospital, Third Military Medical University, 183 Xinqiaozheng Street, Shapingba District, Chongqing, 400037, China.

*** Correspondence Addressed to:**
Hongting Zheng, M.D.
Department of Endocrinology, Xinqiao Hospital
Third Military Medical University, Chongqing 400037, China
E-mail: fnf7703@hotmail.com, Phone: +86 02368755709, Fax: +86 02368755707

[†] Dr Qu, Y. Zheng, and Wang contributed equally to this work.

# Supplementary Online Content

**eAppendix 1.** Literature Search Strategy

**eFigure 1.** Initial analysis of Other Cancer Risks in Patients with DM Receiving Pioglitazone Versus Control from OB Studies

**eFigure 2.** Bladder Cancer Risks Related to Pioglitazone Use Versus Control for Patients with DM in Global from OB Studies plus RCTs

**eFigure 3.** Subgroup Analyses of Bladder Cancer Risk Related to Pioglitazone Use Versus Control for Patients with DM in Global from OB Studies plus RCTs

**eFigure 4.** Subgroup Analyses of Bladder Cancer Risk Related to Pioglitazone Use Versus Control for Patients with DM in America plus Asia and Europe Separately, from OB Studies plus RCTs

**eFigure 5.** Other Cancer Risks in Patients with DM Receiving Pioglitazone Versus Control from OB Studies plus RCTs

**eFigure 6.** Egger's Test for Publication Bias Analysis Regarding Bladder Cancer Risk and Pioglitazone Use

**eFigure 7.** Begg's Test for Publication Bias Analysis Regarding Bladder Cancer Risk and Pioglitazone Use

**eFigure 8.** HbA1c-Adjustment Subgroup Analyses of Bladder Cancer Risk Related to Pioglitazone Use Versus Control for Patients with DM in Global from OB Studies

**eFigure 9.** HbA1c-Adjustment Subgroup Analyses of Bladder Cancer Risk Related to Pioglitazone Use Versus Control for Patients with DM in America plus Asia and Europe Separately, from OB Studies

**eTable 1.** Characteristics of Studies Regarding Bladder Cancer Risk and Pioglitazone Use

**eTable 2.** Quality Assessment of Studies Regarding Bladder Cancer Risk and Pioglitazone Use

**eReferences.** For eFigure 1, 2, 5, 8 and 9, eTable 1 and 2

**MOOSE Checklist**

## Supplement Figure Legends

**eFigure 1.** Initial Analysis of Other Cancer Risks in Patients with DM Receiving Pioglitazone Versus Control from OB Studies[a]

Abbreviations: DM, diabetes mellitus; OB, observational studies.

[a] With Vallarino et al, 2013 included.

**eFigure 2.** Bladder Cancer Risks Related to Pioglitazone Use Versus Control for Patients with DM in Global from OB Studies plus RCTs

Abbreviations: RCTs, randomized controlled trials.

[a] Given that both the exposed and control groups did not report bladder cancer incidence, the OR was not estimable.

**eFigure 3.** Subgroup Analyses of Bladder Cancer Risk Related to Pioglitazone Use Versus Control for Patients with DM in Global from OB Studies plus RCTs

Abbreviations: T2DM, type 2 diabetes mellitus; T1DM, type 1 diabetes mellitus.

[a] With T1DM excluded.

[b] With T1DM included.

**eFigure 4.** Subgroup Analyses of Bladder Cancer Risk Related to Pioglitazone Use Versus Control for Patients with DM in Europe, and America plus Asia, Separately, from OB Studies plus RCTs

[a] With T1DM excluded.

[b] With T1DM included.

**eFigure 5.** Other Cancer Risks in Patients with DM Receiving Pioglitazone Versus Control from OB Studies plus RCTs[a]

[a] With Vallarino et al, 2013 excluded.

**eFigure 6.** Egger's Test for Publication Bias Analysis Regarding Bladder Cancer Risk and Pioglitazone Use

**eFigure 7.** Begg's Test for Publication Bias Analysis Regarding Bladder Cancer Risk and Pioglitazone Use

**eFigure 8.** HbA1c-Adjustment Subgroup Analyses of Bladder Cancer Risk Related to Pioglitazone Use Versus Control for Patients with DM in Global from OB Studies

Abbreviations: HbA1c, hemoglobin A1c concentration.

[a] Given that both the exposed and control groups did not report bladder cancer case, the OR was not estimable.

**eFigure 9.** HbA1c-Adjustment Subgroup Analyses of Bladder Cancer Risk Related to Pioglitazone Use Versus Control

for Patients with DM in America plus Asia and Europe Separately, from OB Studies

[a] Given that both the exposed and control groups did not report bladder cancer case, the OR was not estimable.

**eAppendix 1.** Literature Search Strategy

1) Following search strategy was used: PPAR or peroxisome proliferator activated receptor agonist* or peroxisome proliferator-activated receptor agonist* OR peroxisome proliferator activated receptor activator* OR peroxisome proliferator-activated receptor activator* OR thiazolidinedione*/exp OR thiazolidinedione* OR TZD OR TZDs OR pioglitazone/exp OR pioglitazone OR actos/exp OR actos) AND (cancer/exp OR cancer OR tumor/exp OR tumor OR carcinoma/exp OR carcinoma OR neoplasm/exp OR neoplasm OR malignancy)))) NOT ((((((PPAR or peroxisome proliferator activated receptor agonist* or peroxisome proliferator-activated receptor agonist* OR peroxisome proliferator activated receptor activator* OR peroxisome proliferator-activated receptor activator* OR thiazolidinedione*/exp OR thiazolidinedione* OR TZD OR TZDs OR pioglitazone/exp OR pioglitazone OR actos/exp OR actos) AND (cancer/exp OR cancer OR tumor/exp OR tumor OR carcinoma/exp OR carcinoma OR neoplasm/exp OR neoplasm OR malignancy))) AND Animals [Mesh:noexp])) NOT ((((PPAR or peroxisome proliferator activated receptor agonist* or peroxisome proliferator-activated receptor agonist* OR peroxisome proliferator activated receptor activator* OR peroxisome proliferator-activated receptor activator* OR thiazolidinedione*/exp OR thiazolidinedione* OR TZD OR TZDs OR pioglitazone/exp OR pioglitazone OR actos/exp OR actos) AND (cancer/exp OR cancer OR tumor/exp OR tumor OR carcinoma/exp OR carcinoma OR neoplasm/exp OR neoplasm OR malignancy))) AND Humans[Mesh]))

2) For **Cochrane Central Register of Controlled Trials (CENTRAL)** following search strategy was used: (PPAR or peroxisome proliferator activated receptor agonist* or peroxisome proliferator-activated receptor agonist* or peroxisome proliferator activated receptor activator* or peroxisome proliferator-activated receptor activator* or thiazolidinedione* or TZD* or pioglitazone or Actos) AND (cancer* or tumor* or carcinoma* or neoplasm* or malignancy*)

3) For **ClinicalTrials.gov** following search strategy was used: "thiazolidinediones" OR "TZDs" OR "pioglitazone" OR "Actos" | Studies With Results (We did not use the general terms to search the ClinicalTrails.gov, such as “PPAR” and “peroxisome proliferator activated receptor agonist”, as this registry is designed so that one can capture relevant trials using generic drug names directly.)

eFigure 1

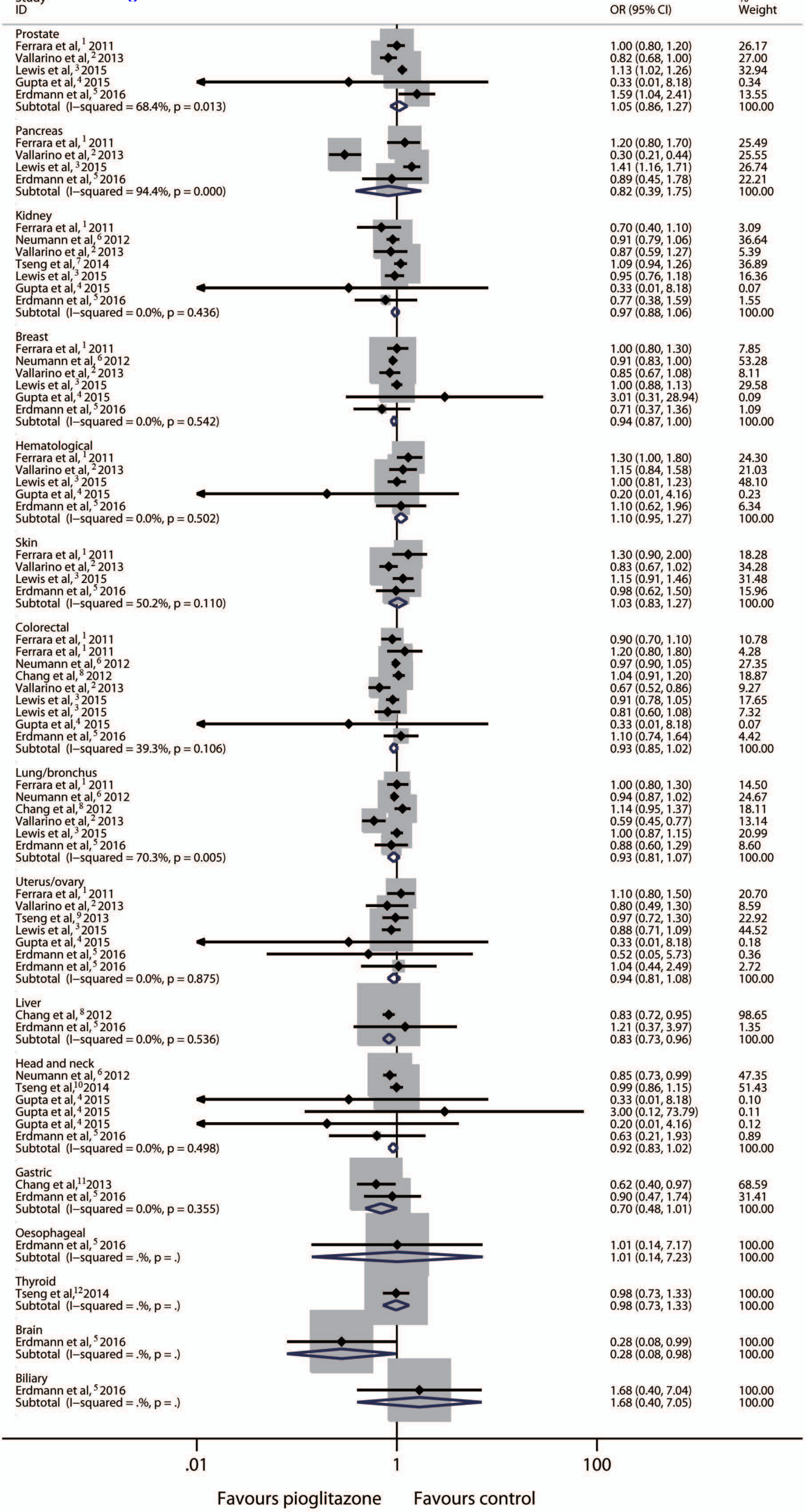
Study ID
OR (95% CI)
% Weight
Prostate
Ferrara et al,[1] 2011 1.00 (0.80, 1.20) 26.17
Vallarino et al,[2] 2013 0.82 (0.68, 1.00) 27.00
Lewis et al,[3] 2015 1.13 (1.02, 1.26) 32.94
Gupta et al,[4] 2015 0.33 (0.01, 8.18) 0.34
Erdmann et al,[5] 2016 1.59 (1.04, 2.41) 13.55
Subtotal (I−squared = 68.4%, p = 0.013) 1.05 (0.86, 1.27) 100.00
Pancreas
Ferrara et al,[1] 2011 1.20 (0.80, 1.70) 25.49
Vallarino et al,[2] 2013 0.30 (0.21, 0.44) 25.55
Lewis et al,[3] 2015 1.41 (1.16, 1.71) 26.74
Erdmann et al,[5] 2016 0.89 (0.45, 1.78) 22.21
Subtotal (I−squared = 94.4%, p = 0.000) 0.82 (0.39, 1.75) 100.00
Kidney
Ferrara et al,[1] 2011 0.70 (0.40, 1.10) 3.09
Neumann et al,[6] 2012 0.91 (0.79, 1.06) 36.64
Vallarino et al,[2] 2013 0.87 (0.59, 1.27) 5.39
Tseng et al,[7] 2014 1.09 (0.94, 1.26) 36.89
Lewis et al,[3] 2015 0.95 (0.76, 1.18) 16.36
Gupta et al,[4] 2015 0.33 (0.01, 8.18) 0.07
Erdmann et al,[5] 2016 0.77 (0.38, 1.59) 1.55
Subtotal (I−squared = 0.0%, p = 0.436) 0.97 (0.88, 1.06) 100.00
Breast
Ferrara et al,[1] 2011 1.00 (0.80, 1.30) 7.85
Neumann et al,[6] 2012 0.91 (0.83, 1.00) 53.28
Vallarino et al,[2] 2013 0.85 (0.67, 1.08) 8.11
Lewis et al,[3] 2015 1.00 (0.88, 1.13) 29.58
Gupta et al,[4] 2015 3.01 (0.31, 28.94) 0.09
Erdmann et al,[5] 2016 0.71 (0.37, 1.36) 1.09
Subtotal (I−squared = 0.0%, p = 0.542) 0.94 (0.87, 1.00) 100.00
Hematological
Ferrara et al,[1] 2011 1.30 (1.00, 1.80) 24.30
Vallarino et al,[2] 2013 1.15 (0.84, 1.58) 21.03
Lewis et al,[3] 2015 1.00 (0.81, 1.23) 48.10
Gupta et al,[4] 2015 0.20 (0.01, 4.16) 0.23
Erdmann et al,[5] 2016 1.10 (0.62, 1.96) 6.34
Subtotal (I−squared = 0.0%, p = 0.502) 1.10 (0.95, 1.27) 100.00
Skin
Ferrara et al,[1] 2011 1.30 (0.90, 2.00) 18.28
Vallarino et al,[2] 2013 0.83 (0.67, 1.02) 34.28
Lewis et al,[3] 2015 1.15 (0.91, 1.46) 31.48
Erdmann et al,[5] 2016 0.98 (0.62, 1.50) 15.96
Subtotal (I−squared = 50.2%, p = 0.110) 1.03 (0.83, 1.27) 100.00
Colorectal
Ferrara et al,[1] 2011 0.90 (0.70, 1.10) 10.78
Ferrara et al,[1] 2011 1.20 (0.80, 1.80) 4.28
Neumann et al,[6] 2012 0.97 (0.90, 1.05) 27.35
Chang et al,[8] 2012 1.04 (0.91, 1.20) 18.87
Vallarino et al,[2] 2013 0.67 (0.52, 0.86) 9.27
Lewis et al,[3] 2015 0.91 (0.78, 1.05) 17.65
Lewis et al,[3] 2015 0.81 (0.60, 1.08) 7.32
Gupta et al,[4] 2015 0.33 (0.01, 8.18) 0.07
Erdmann et al,[5] 2016 1.10 (0.74, 1.64) 4.42
Subtotal (I−squared = 39.3%, p = 0.106) 0.93 (0.85, 1.02) 100.00
Lung/bronchus
Ferrara et al,[1] 2011 1.00 (0.80, 1.30) 14.50
Neumann et al,[6] 2012 0.94 (0.87, 1.02) 24.67
Chang et al,[8] 2012 1.14 (0.95, 1.37) 18.11
Vallarino et al,[2] 2013 0.59 (0.45, 0.77) 13.14
Lewis et al,[3] 2015 1.00 (0.87, 1.15) 20.99
Erdmann et al,[5] 2016 0.88 (0.60, 1.29) 8.60
Subtotal (I−squared = 70.3%, p = 0.005) 0.93 (0.81, 1.07) 100.00
Uterus/ovary
Ferrara et al,[1] 2011 1.10 (0.80, 1.50) 20.70
Vallarino et al,[2] 2013 0.80 (0.49, 1.30) 8.59
Tseng et al,[9] 2013 0.97 (0.72, 1.30) 22.92
Lewis et al,[3] 2015 0.88 (0.71, 1.09) 44.52
Gupta et al,[4] 2015 0.33 (0.01, 8.18) 0.18
Erdmann et al,[5] 2016 0.52 (0.05, 5.73) 0.36
Erdmann et al,[5] 2016 1.04 (0.44, 2.49) 2.72
Subtotal (I−squared = 0.0%, p = 0.875) 0.94 (0.81, 1.08) 100.00
Liver
Chang et al,[8] 2012 0.83 (0.72, 0.95) 98.65
Erdmann et al,[5] 2016 1.21 (0.37, 3.97) 1.35
Subtotal (I−squared = 0.0%, p = 0.536) 0.83 (0.73, 0.96) 100.00
Head and neck
Neumann et al,[6] 2012 0.85 (0.73, 0.99) 47.35
Tseng et al,[10] 2014 0.99 (0.86, 1.15) 51.43
Gupta et al,[4] 2015 0.33 (0.01, 8.18) 0.10
Gupta et al,[4] 2015 3.00 (0.12, 73.79) 0.11
Gupta et al,[4] 2015 0.20 (0.01, 4.16) 0.12
Erdmann et al,[5] 2016 0.63 (0.21, 1.93) 0.89
Subtotal (I−squared = 0.0%, p = 0.498) 0.92 (0.83, 1.02) 100.00
Gastric
Chang et al,[11] 2013 0.62 (0.40, 0.97) 68.59
Erdmann et al,[5] 2016 0.90 (0.47, 1.74) 31.41
Subtotal (I−squared = 0.0%, p = 0.355) 0.70 (0.48, 1.01) 100.00
Oesophageal
Erdmann et al,[5] 2016 1.01 (0.14, 7.17) 100.00
Subtotal (I−squared = .%, p = .) 1.01 (0.14, 7.23) 100.00
Thyroid
Tseng et al,[12] 2014 0.98 (0.73, 1.33) 100.00
Subtotal (I−squared = .%, p = .) 0.98 (0.73, 1.33) 100.00
Brain
Erdmann et al,[5] 2016 0.28 (0.08, 0.99) 100.00
Subtotal (I−squared = .%, p = .) 0.28 (0.08, 0.98) 100.00
Biliary
Erdmann et al,[5] 2016 1.68 (0.40, 7.04) 100.00
Subtotal (I−squared = .%, p = .) 1.68 (0.40, 7.05) 100.00
.01
1
100
Favours pioglitazone
Favours control

eFigure 2

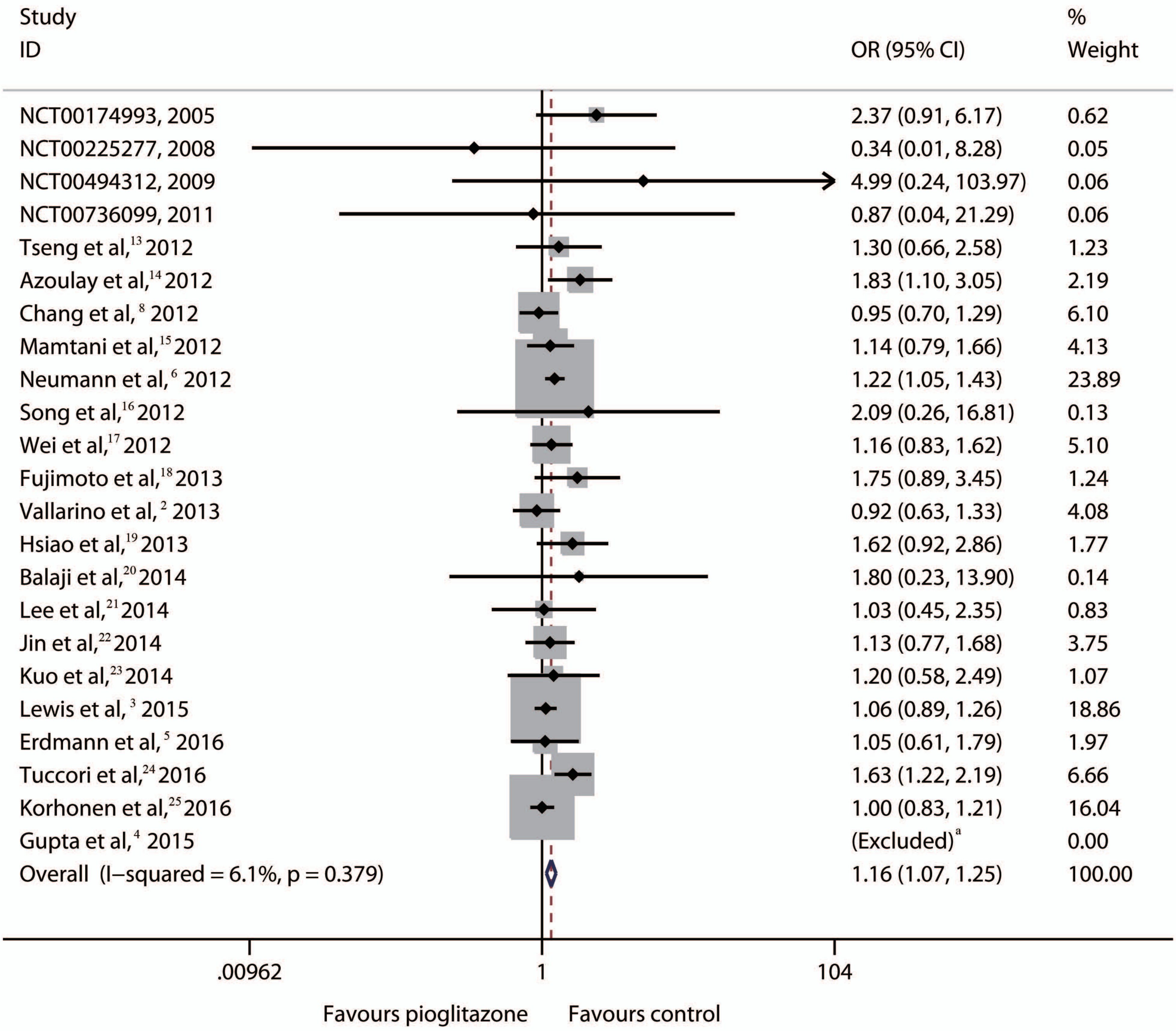
Study
ID
OR (95% CI)
%
Weight
NCT00174993, 2005
2.37 (0.91, 6.17)
0.62
NCT00225277, 2008
0.34 (0.01, 8.28)
0.05
NCT00494312, 2009
4.99 (0.24, 103.97)
0.06
NCT00736099, 2011
0.87 (0.04, 21.29)
0.06
Tseng et al,[13] 2012
1.30 (0.66, 2.58)
1.23
Azoulay et al,[14] 2012
1.83 (1.10, 3.05)
2.19
Chang et al,[8] 2012
0.95 (0.70, 1.29)
6.10
Mamtani et al,[15] 2012
1.14 (0.79, 1.66)
4.13
Neumann et al,[6] 2012
1.22 (1.05, 1.43)
23.89
Song et al,[16] 2012
2.09 (0.26, 16.81)
0.13
Wei et al,[17] 2012
1.16 (0.83, 1.62)
5.10
Fujimoto et al,[18] 2013
1.75 (0.89, 3.45)
1.24
Vallarino et al,[2] 2013
0.92 (0.63, 1.33)
4.08
Hsiao et al,[19] 2013
1.62 (0.92, 2.86)
1.77
Balaji et al,[20] 2014
1.80 (0.23, 13.90)
0.14
Lee et al,[21] 2014
1.03 (0.45, 2.35)
0.83
Jin et al,[22] 2014
1.13 (0.77, 1.68)
3.75
Kuo et al,[23] 2014
1.20 (0.58, 2.49)
1.07
Lewis et al,[3] 2015
1.06 (0.89, 1.26)
18.86
Erdmann et al,[5] 2016
1.05 (0.61, 1.79)
1.97
Tuccori et al,[24] 2016
1.63 (1.22, 2.19)
6.66
Korhonen et al,[25] 2016
1.00 (0.83, 1.21)
16.04
Gupta et al,[4] 2015
(Excluded)[a]
0.00
Overall (I–squared = 6.1%, p = 0.379)
1.16 (1.07, 1.25)
100.00
.00962
1
104
Favours pioglitazone
Favours control

eFigure 3

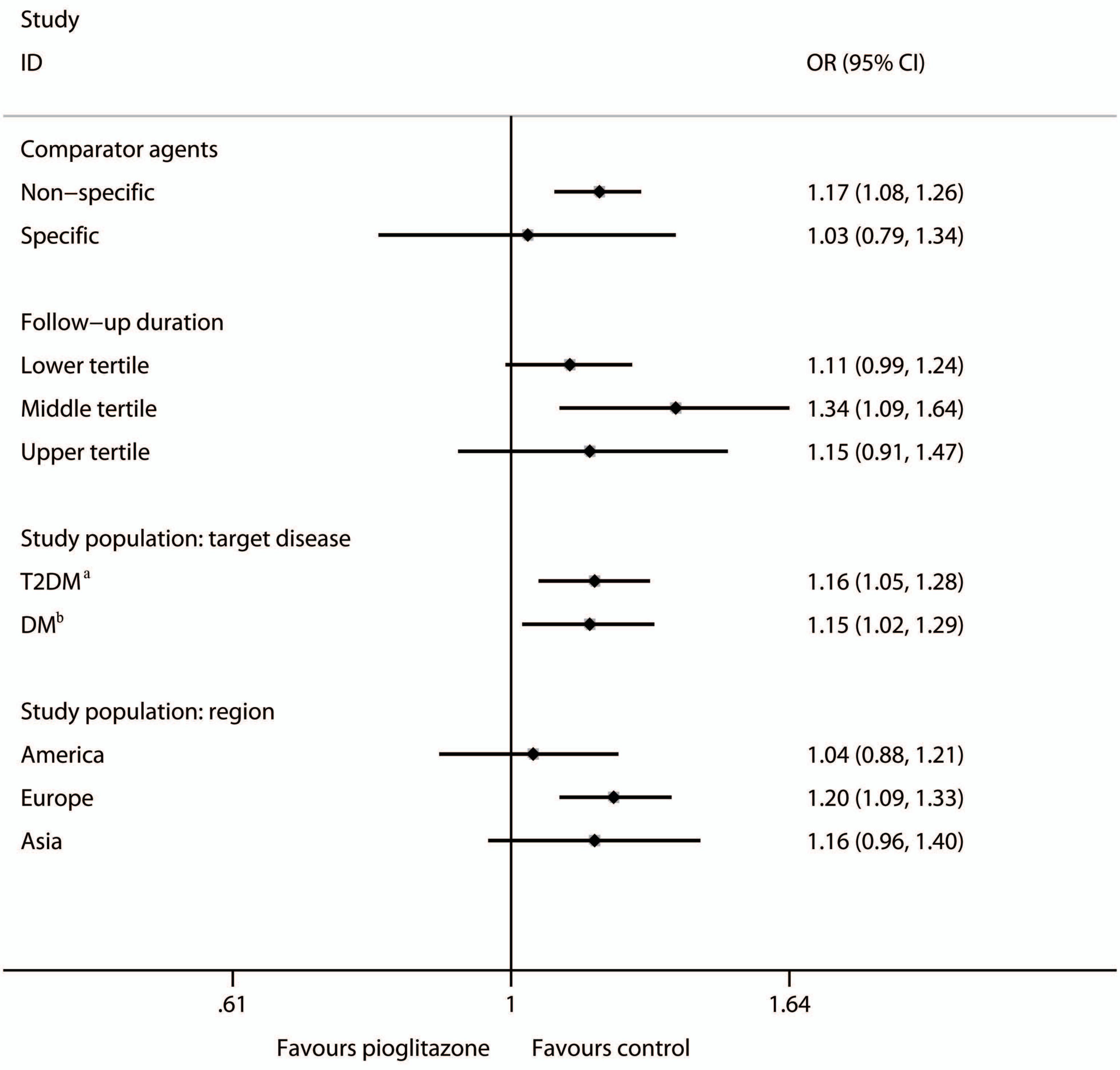
Study
ID
OR (95% CI)
Comparator agents
Non–specific
1.17 (1.08, 1.26)
Specific
1.03 (0.79, 1.34)
Follow–up duration
Lower tertile
1.11 (0.99, 1.24)
Middle tertile
1.34 (1.09, 1.64)
Upper tertile
1.15 (0.91, 1.47)
Study population: target disease
T2DM[a]
1.16 (1.05, 1.28)
DM[b]
1.15 (1.02, 1.29)
Study population: region
America
1.04 (0.88, 1.21)
Europe
1.20 (1.09, 1.33)
Asia
1.16 (0.96, 1.40)
.61
1
1.64
Favours pioglitazone
Favours control

eFigure 4

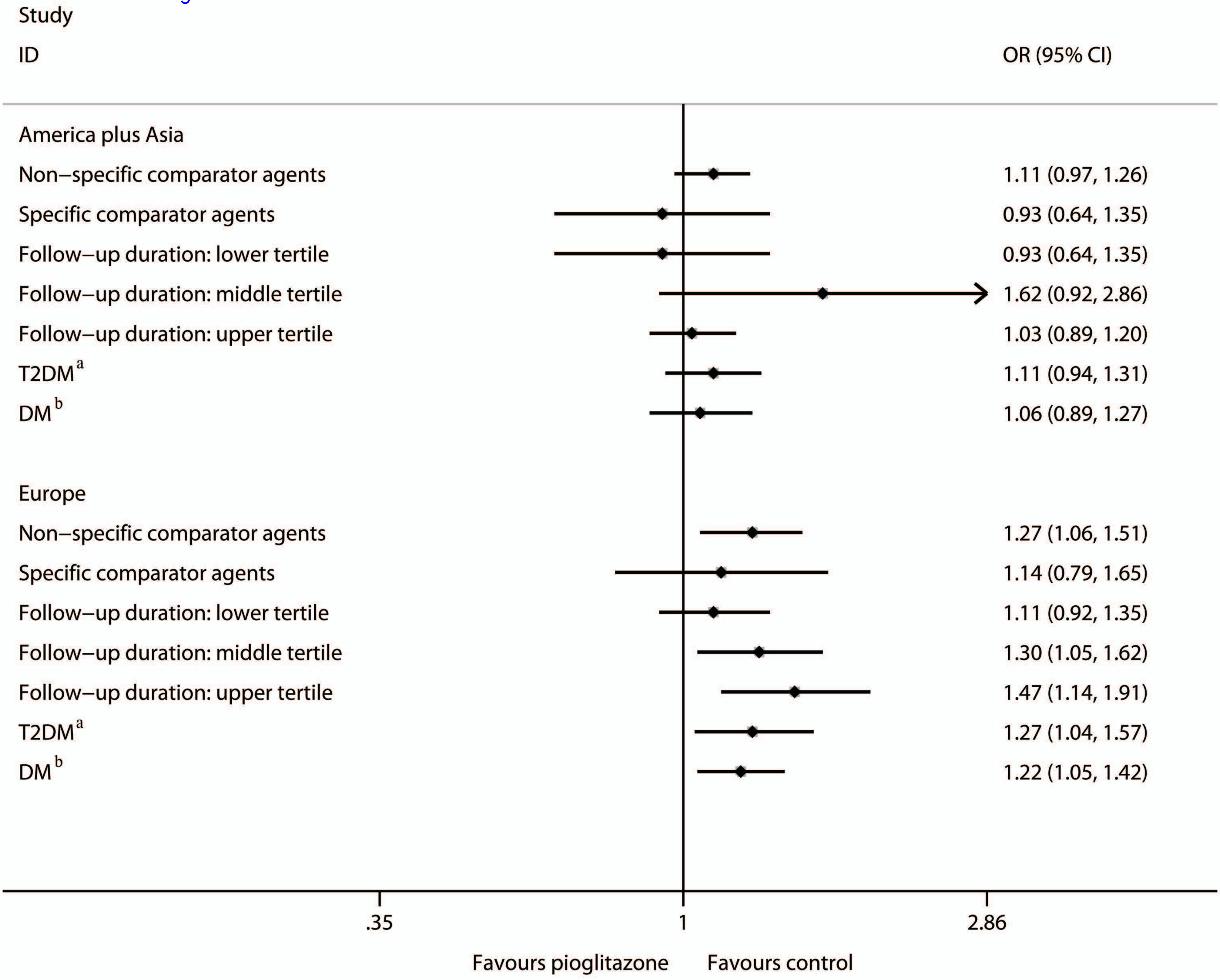
Study
ID
OR (95% CI)
America plus Asia
Non–specific comparator agents
1.11 (0.97, 1.26)
Specific comparator agents
0.93 (0.64, 1.35)
Follow–up duration: lower tertile
0.93 (0.64, 1.35)
Follow–up duration: middle tertile
1.62 (0.92, 2.86)
Follow–up duration: upper tertile
1.03 (0.89, 1.20)
T2DM a
1.11 (0.94, 1.31)
DM b
1.06 (0.89, 1.27)
Europe
Non–specific comparator agents
1.27 (1.06, 1.51)
Specific comparator agents
1.14 (0.79, 1.65)
Follow–up duration: lower tertile
1.11 (0.92, 1.35)
Follow–up duration: middle tertile
1.30 (1.05, 1.62)
Follow–up duration: upper tertile
1.47 (1.14, 1.91)
T2DM a
1.27 (1.04, 1.57)
DM b
1.22 (1.05, 1.42)
.35
1
2.86
Favours pioglitazone
Favours control

eFigure 5

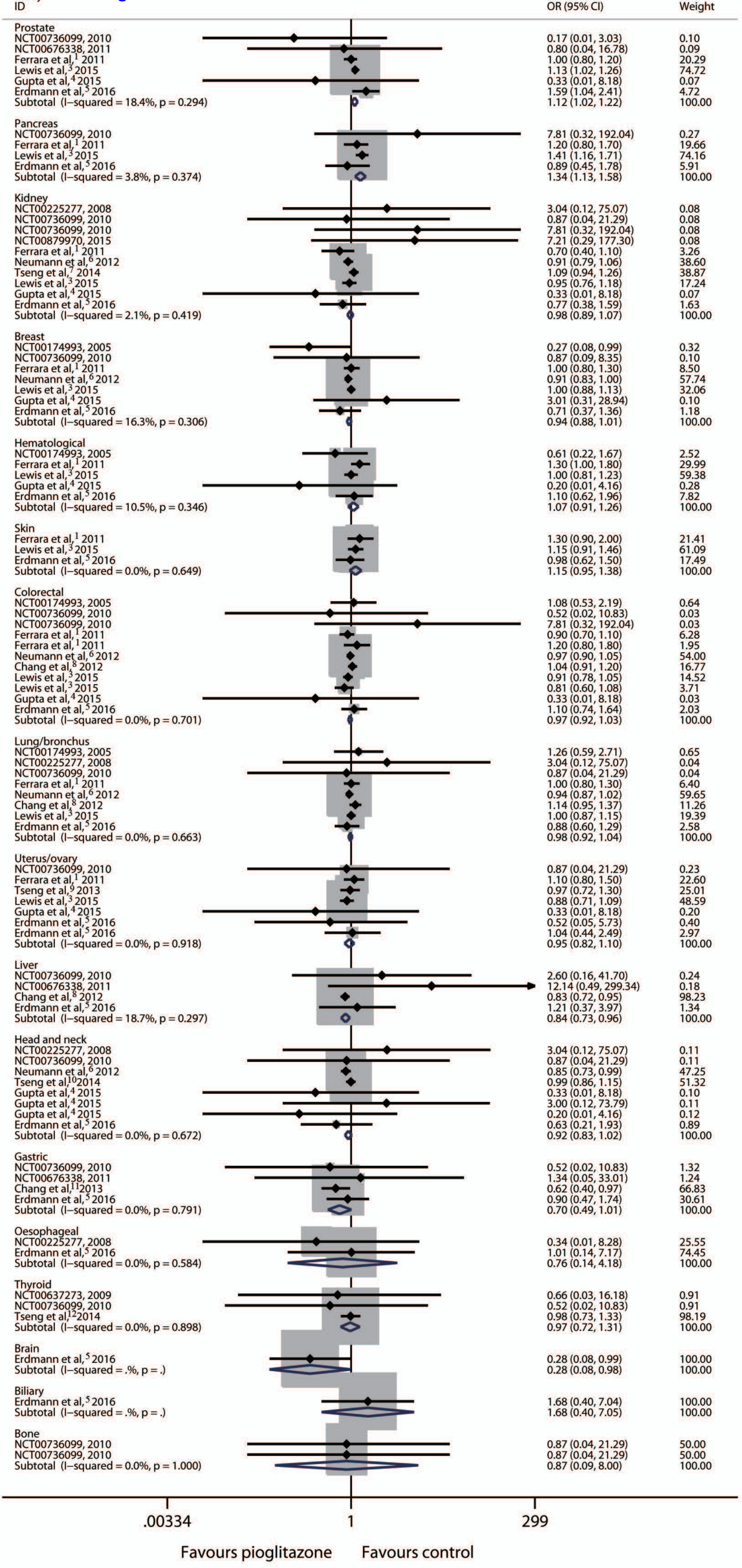

Study ID | OR (95% CI) | % Weight
Prostate
NCT00736099, 2010 | 0.17 (0.01, 3.03) | 0.10
NCT00676338, 2011 | 0.80 (0.04, 16.78) | 0.09
Ferrara et al,[1] 2011 | 1.00 (0.80, 1.20) | 20.29
Lewis et al,[3] 2015 | 1.13 (1.02, 1.26) | 74.72
Gupta et al,[4] 2015 | 0.33 (0.01, 8.18) | 0.07
Erdmann et al,[5] 2016 | 1.59 (1.04, 2.41) | 4.72
Subtotal (I−squared = 18.4%, p = 0.294) | 1.12 (1.02, 1.22) | 100.00
Pancreas
NCT00736099, 2010 | 7.81 (0.32, 192.04) | 0.27
Ferrara et al,[1] 2011 | 1.20 (0.80, 1.70) | 19.66
Lewis et al,[3] 2015 | 1.41 (1.16, 1.71) | 74.16
Erdmann et al,[5] 2016 | 0.89 (0.45, 1.78) | 5.91
Subtotal (I−squared = 3.8%, p = 0.374) | 1.34 (1.13, 1.58) | 100.00
Kidney
NCT00225277, 2008 | 3.04 (0.12, 75.07) | 0.08
NCT00736099, 2010 | 0.87 (0.04, 21.29) | 0.08
NCT00736099, 2010 | 7.81 (0.32, 192.04) | 0.08
NCT00879970, 2015 | 7.21 (0.29, 177.30) | 0.08
Ferrara et al,[1] 2011 | 0.70 (0.40, 1.10) | 3.26
Neumann et al,[6] 2012 | 0.91 (0.79, 1.06) | 38.60
Tseng et al,[7] 2014 | 1.09 (0.94, 1.26) | 38.87
Lewis et al,[3] 2015 | 0.95 (0.76, 1.18) | 17.24
Gupta et al,[4] 2015 | 0.33 (0.01, 8.18) | 0.07
Erdmann et al,[5] 2016 | 0.77 (0.38, 1.59) | 1.63
Subtotal (I−squared = 2.1%, p = 0.419) | 0.98 (0.89, 1.07) | 100.00
Breast
NCT00174993, 2005 | 0.27 (0.08, 0.99) | 0.32
NCT00736099, 2010 | 0.87 (0.09, 8.35) | 0.10
Ferrara et al,[1] 2011 | 1.00 (0.80, 1.30) | 8.50
Neumann et al,[6] 2012 | 0.91 (0.83, 1.00) | 57.74
Lewis et al,[3] 2015 | 1.00 (0.88, 1.13) | 32.06
Gupta et al,[4] 2015 | 3.01 (0.31, 28.94) | 0.10
Erdmann et al,[5] 2016 | 0.71 (0.37, 1.36) | 1.18
Subtotal (I−squared = 16.3%, p = 0.306) | 0.94 (0.88, 1.01) | 100.00
Hematological
NCT00174993, 2005 | 0.61 (0.22, 1.67) | 2.52
Ferrara et al,[1] 2011 | 1.30 (1.00, 1.80) | 29.99
Lewis et al,[3] 2015 | 1.00 (0.81, 1.23) | 59.38
Gupta et al,[4] 2015 | 0.20 (0.01, 4.16) | 0.28
Erdmann et al,[5] 2016 | 1.10 (0.62, 1.96) | 7.82
Subtotal (I−squared = 10.5%, p = 0.346) | 1.07 (0.91, 1.26) | 100.00
Skin
Ferrara et al,[1] 2011 | 1.30 (0.90, 2.00) | 21.41
Lewis et al,[3] 2015 | 1.15 (0.91, 1.46) | 61.09
Erdmann et al,[5] 2016 | 0.98 (0.62, 1.50) | 17.49
Subtotal (I−squared = 0.0%, p = 0.649) | 1.15 (0.95, 1.38) | 100.00
Colorectal
NCT00174993, 2005 | 1.08 (0.53, 2.19) | 0.64
NCT00736099, 2010 | 0.52 (0.02, 10.83) | 0.03
NCT00736099, 2010 | 7.81 (0.32, 192.04) | 0.03
Ferrara et al,[1] 2011 | 0.90 (0.70, 1.10) | 6.28
Ferrara et al,[1] 2011 | 1.20 (0.80, 1.80) | 1.95
Neumann et al,[6] 2012 | 0.97 (0.90, 1.05) | 54.00
Chang et al,[8] 2012 | 1.04 (0.91, 1.20) | 16.77
Lewis et al,[3] 2015 | 0.91 (0.78, 1.05) | 14.52
Lewis et al,[3] 2015 | 0.81 (0.60, 1.08) | 3.71
Gupta et al,[4] 2015 | 0.33 (0.01, 8.18) | 0.03
Erdmann et al,[5] 2016 | 1.10 (0.74, 1.64) | 2.03
Subtotal (I−squared = 0.0%, p = 0.701) | 0.97 (0.92, 1.03) | 100.00
Lung/bronchus
NCT00174993, 2005 | 1.26 (0.59, 2.71) | 0.65
NCT00225277, 2008 | 3.04 (0.12, 75.07) | 0.04
NCT00736099, 2010 | 0.87 (0.04, 21.29) | 0.04
Ferrara et al,[1] 2011 | 1.00 (0.80, 1.30) | 6.40
Neumann et al,[6] 2012 | 0.94 (0.87, 1.02) | 59.65
Chang et al,[8] 2012 | 1.14 (0.95, 1.37) | 11.26
Lewis et al,[3] 2015 | 1.00 (0.87, 1.15) | 19.39
Erdmann et al,[5] 2016 | 0.88 (0.60, 1.29) | 2.58
Subtotal (I−squared = 0.0%, p = 0.663) | 0.98 (0.92, 1.04) | 100.00
Uterus/ovary
NCT00736099, 2010 | 0.87 (0.04, 21.29) | 0.23
Ferrara et al,[1] 2011 | 1.10 (0.80, 1.50) | 22.60
Tseng et al,[9] 2013 | 0.97 (0.72, 1.30) | 25.01
Lewis et al,[3] 2015 | 0.88 (0.71, 1.09) | 48.59
Gupta et al,[4] 2015 | 0.33 (0.01, 8.18) | 0.20
Erdmann et al,[5] 2016 | 0.52 (0.05, 5.73) | 0.40
Erdmann et al,[5] 2016 | 1.04 (0.44, 2.49) | 2.97
Subtotal (I−squared = 0.0%, p = 0.918) | 0.95 (0.82, 1.10) | 100.00
Liver
NCT00736099, 2010 | 2.60 (0.16, 41.70) | 0.24
NCT00676338, 2011 | 12.14 (0.49, 299.34) | 0.18
Chang et al,[8] 2012 | 0.83 (0.72, 0.95) | 98.23
Erdmann et al,[5] 2016 | 1.21 (0.37, 3.97) | 1.34
Subtotal (I−squared = 18.7%, p = 0.297) | 0.84 (0.73, 0.96) | 100.00
Head and neck
NCT00225277, 2008 | 3.04 (0.12, 75.07) | 0.11
NCT00736099, 2010 | 0.87 (0.04, 21.29) | 0.11
Neumann et al,[6] 2012 | 0.85 (0.73, 0.99) | 47.25
Tseng et al,[10] 2014 | 0.99 (0.86, 1.15) | 51.32
Gupta et al,[4] 2015 | 0.33 (0.01, 8.18) | 0.10
Gupta et al,[4] 2015 | 3.00 (0.12, 73.79) | 0.11
Gupta et al,[4] 2015 | 0.20 (0.01, 4.16) | 0.12
Erdmann et al,[5] 2016 | 0.63 (0.21, 1.93) | 0.89
Subtotal (I−squared = 0.0%, p = 0.672) | 0.92 (0.83, 1.02) | 100.00
Gastric
NCT00736099, 2010 | 0.52 (0.02, 10.83) | 1.32
NCT00676338, 2011 | 1.34 (0.05, 33.01) | 1.24
Chang et al,[11] 2013 | 0.62 (0.40, 0.97) | 66.83
Erdmann et al,[5] 2016 | 0.90 (0.47, 1.74) | 30.61
Subtotal (I−squared = 0.0%, p = 0.791) | 0.70 (0.49, 1.01) | 100.00
Oesophageal
NCT00225277, 2008 | 0.34 (0.01, 8.28) | 25.55
Erdmann et al,[5] 2016 | 1.01 (0.14, 7.17) | 74.45
Subtotal (I−squared = 0.0%, p = 0.584) | 0.76 (0.14, 4.18) | 100.00
Thyroid
NCT00637273, 2009 | 0.66 (0.03, 16.18) | 0.91
NCT00736099, 2010 | 0.52 (0.02, 10.83) | 0.91
Tseng et al,[12] 2014 | 0.98 (0.73, 1.33) | 98.19
Subtotal (I−squared = 0.0%, p = 0.898) | 0.97 (0.72, 1.31) | 100.00
Brain
Erdmann et al,[5] 2016 | 0.28 (0.08, 0.99) | 100.00
Subtotal (I−squared = .%, p = .) | 0.28 (0.08, 0.98) | 100.00
Biliary
Erdmann et al,[5] 2016 | 1.68 (0.40, 7.04) | 100.00
Subtotal (I−squared = .%, p = .) | 1.68 (0.40, 7.05) | 100.00
Bone
NCT00736099, 2010 | 0.87 (0.04, 21.29) | 50.00
NCT00736099, 2010 | 0.87 (0.04, 21.29) | 50.00
Subtotal (I−squared = 0.0%, p = 1.000) | 0.87 (0.09, 8.00) | 100.00
.00334
1
299
Favours pioglitazone
Favours control

eFigure 6 Egger's publication bias plot

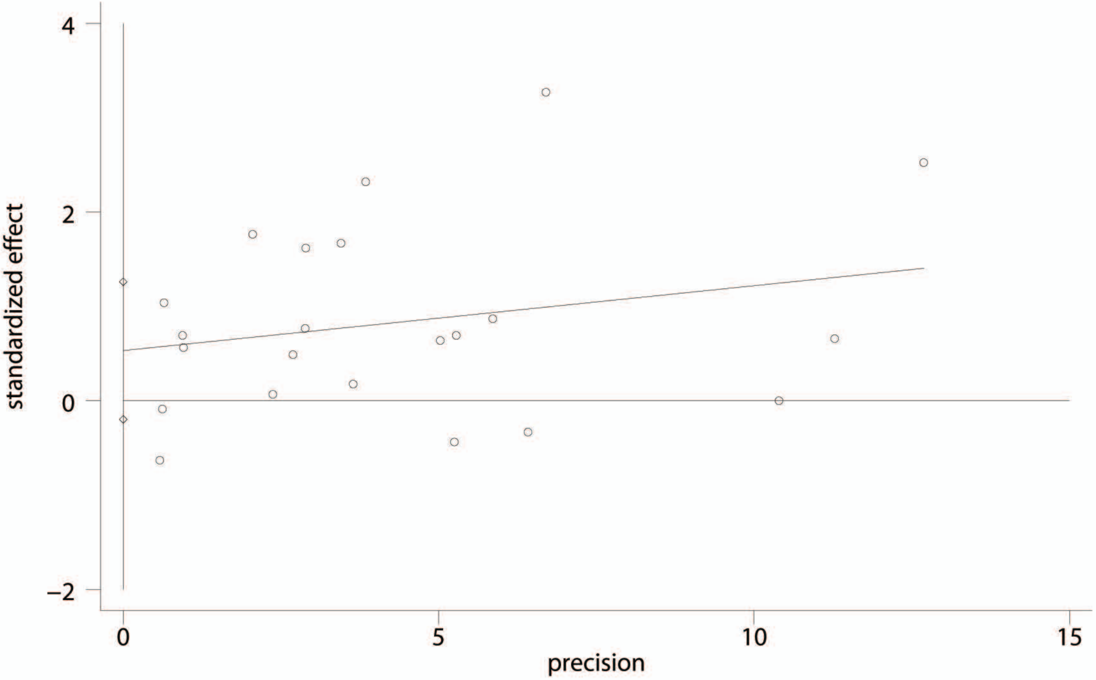

eFigure 7 Begg's funnel plot with pseudo 95% confidence limits

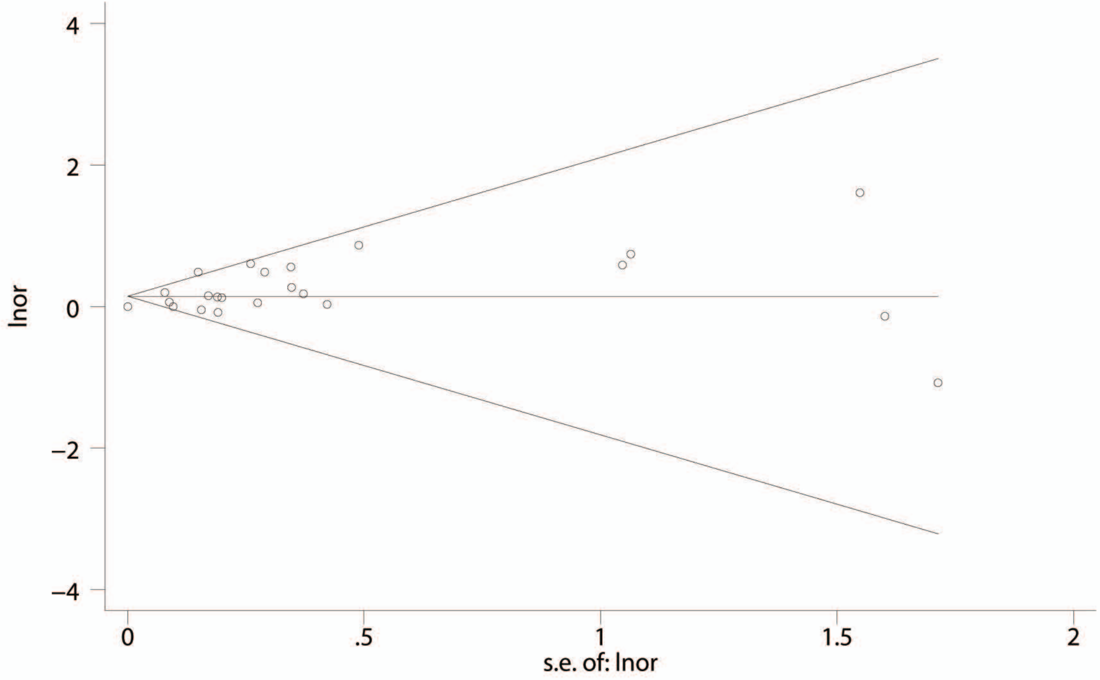

eFigure 8

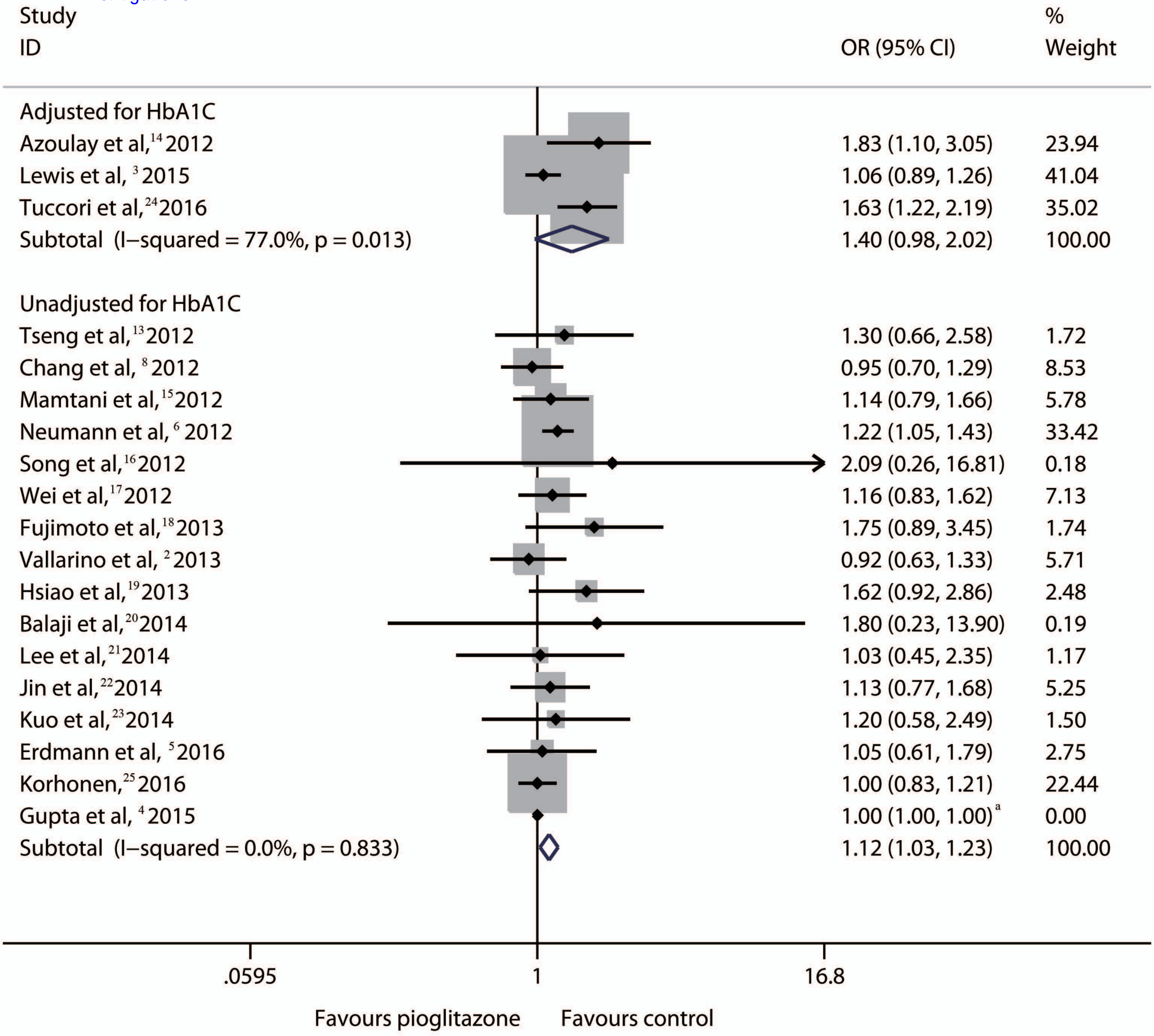
Study
ID
OR (95% CI)
%
Weight
Adjusted for HbA1C
Azoulay et al,[14] 2012 1.83 (1.10, 3.05) 23.94
Lewis et al,[3] 2015 1.06 (0.89, 1.26) 41.04
Tuccori et al,[24] 2016 1.63 (1.22, 2.19) 35.02
Subtotal (I–squared = 77.0%, p = 0.013) 1.40 (0.98, 2.02) 100.00
Unadjusted for HbA1C
Tseng et al,[13] 2012 1.30 (0.66, 2.58) 1.72
Chang et al,[8] 2012 0.95 (0.70, 1.29) 8.53
Mamtani et al,[15] 2012 1.14 (0.79, 1.66) 5.78
Neumann et al,[6] 2012 1.22 (1.05, 1.43) 33.42
Song et al,[16] 2012 2.09 (0.26, 16.81) 0.18
Wei et al,[17] 2012 1.16 (0.83, 1.62) 7.13
Fujimoto et al,[18] 2013 1.75 (0.89, 3.45) 1.74
Vallarino et al,[2] 2013 0.92 (0.63, 1.33) 5.71
Hsiao et al,[19] 2013 1.62 (0.92, 2.86) 2.48
Balaji et al,[20] 2014 1.80 (0.23, 13.90) 0.19
Lee et al,[21] 2014 1.03 (0.45, 2.35) 1.17
Jin et al,[22] 2014 1.13 (0.77, 1.68) 5.25
Kuo et al,[23] 2014 1.20 (0.58, 2.49) 1.50
Erdmann et al,[5] 2016 1.05 (0.61, 1.79) 2.75
Korhonen,[25] 2016 1.00 (0.83, 1.21) 22.44
Gupta et al,[4] 2015 1.00 (1.00, 1.00)[a] 0.00
Subtotal (I–squared = 0.0%, p = 0.833) 1.12 (1.03, 1.23) 100.00
.0595
1
16.8
Favours pioglitazone
Favours control

eFigure 9

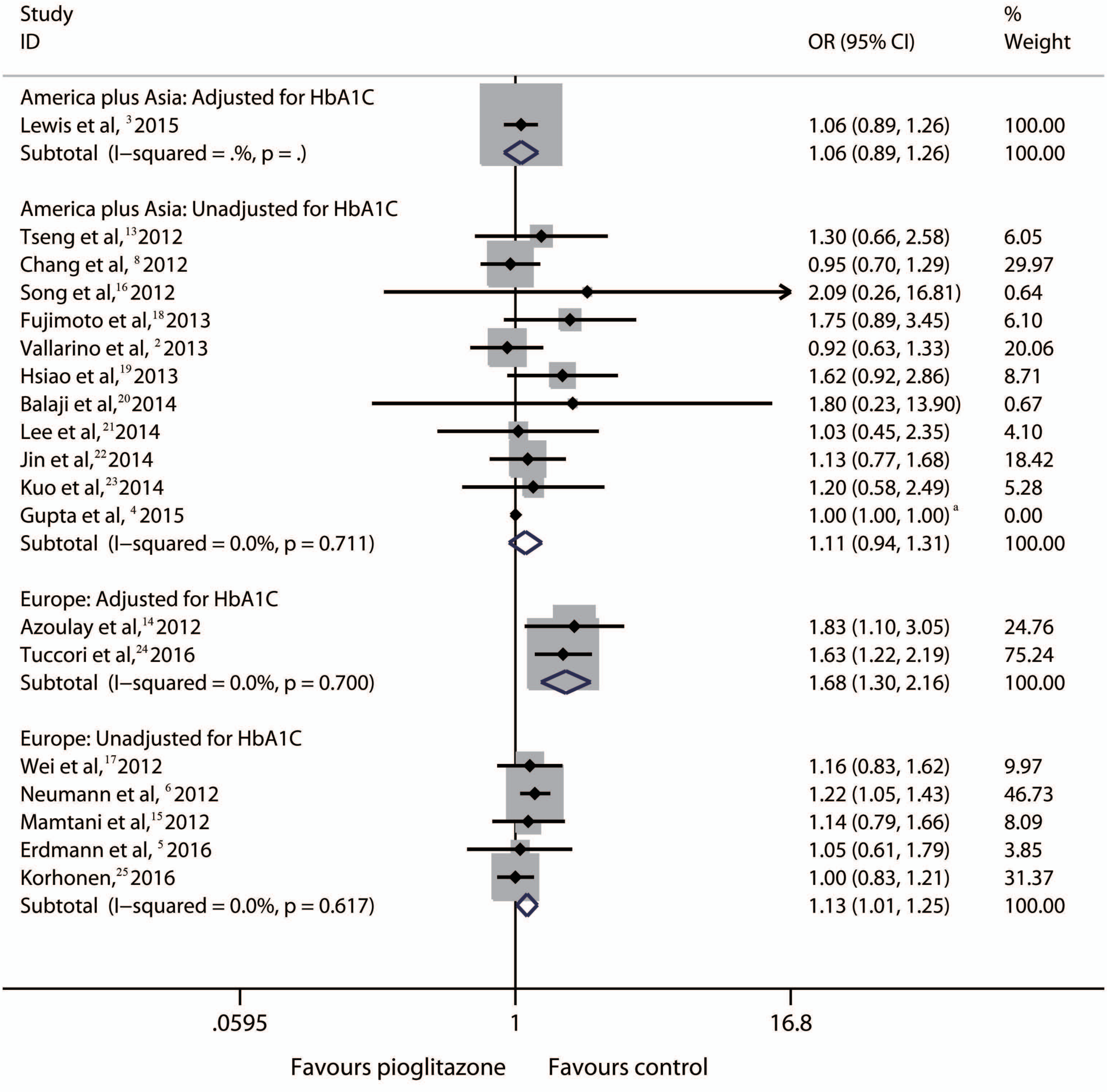

Study
ID
OR (95% CI)
%
Weight
America plus Asia: Adjusted for HbA1C
Lewis et al, [3] 2015
1.06 (0.89, 1.26)
100.00
Subtotal (I–squared = .%, p = .)
1.06 (0.89, 1.26)
100.00
America plus Asia: Unadjusted for HbA1C
Tseng et al, [13] 2012
1.30 (0.66, 2.58)
6.05
Chang et al, [8] 2012
0.95 (0.70, 1.29)
29.97
Song et al, [16] 2012
2.09 (0.26, 16.81)
0.64
Fujimoto et al, [18] 2013
1.75 (0.89, 3.45)
6.10
Vallarino et al, [2] 2013
0.92 (0.63, 1.33)
20.06
Hsiao et al, [19] 2013
1.62 (0.92, 2.86)
8.71
Balaji et al, [20] 2014
1.80 (0.23, 13.90)
0.67
Lee et al, [21] 2014
1.03 (0.45, 2.35)
4.10
Jin et al, [22] 2014
1.13 (0.77, 1.68)
18.42
Kuo et al, [23] 2014
1.20 (0.58, 2.49)
5.28
Gupta et al, [4] 2015
1.00 (1.00, 1.00) [a]
0.00
Subtotal (I–squared = 0.0%, p = 0.711)
1.11 (0.94, 1.31)
100.00
Europe: Adjusted for HbA1C
Azoulay et al, [14] 2012
1.83 (1.10, 3.05)
24.76
Tuccori et al, [24] 2016
1.63 (1.22, 2.19)
75.24
Subtotal (I–squared = 0.0%, p = 0.700)
1.68 (1.30, 2.16)
100.00
Europe: Unadjusted for HbA1C
Wei et al, [17] 2012
1.16 (0.83, 1.62)
9.97
Neumann et al, [6] 2012
1.22 (1.05, 1.43)
46.73
Mamtani et al, [15] 2012
1.14 (0.79, 1.66)
8.09
Erdmann et al, [5] 2016
1.05 (0.61, 1.79)
3.85
Korhonen, [25] 2016
1.00 (0.83, 1.21)
31.37
Subtotal (I–squared = 0.0%, p = 0.617)
1.13 (1.01, 1.25)
100.00
.0595
1
16.8
Favours pioglitazone
Favours control

**eTable 1.** Characteristics of Studies Regarding Bladder Cancer Risk and Pioglitazone Use

| **Author (year)** | **Study Type** | **Region** | **Target disease** | **Mean period of follow-up (years)** | **Dose-response gradient (Y/N)** | **Duration-response gradient (Y/N)** | **Mean age (years)** | **Male patient No. (%)** | **Exposure group** | **Control group** | **No. of pioglitazone (events/ total)** | **No. of control (events/ total)** | **Adjusted estimates (95% CI)** | **Adjusted covariate** |
|---|---|---|---|---|---|---|---|---|---|---|---|---|---|---|
| Tseng et al,[13] (2012) | Cohort | Taiwan | T2DM | NR | Y[a] | Y[a] | NR | NR | Pioglitazone | No use | 10 /2545 | 155 /52383 | HR 1.30 (0.66, 2.58) | Age, sex, diabetes duration, nephropathy, urinary tract disease, hypertension, COPD, cerebrovascular disease, ischemic heart disease, peripheral arterial disease, eye disease, dyslipidemia, heart failure, rosiglitazone, sulfonylurea, meglitinide, metformin, acarbose, insulin, statin, fibrate, ACE inhibitor/angiotensin receptor blocker, calcium channel blocker, region of residence, occupation, and other cancer before baseline. |
| Azoulay et al,[14] (2012) | Nested case/control | UK | T2DM | 4.6 (mean) | Y[a] | Y[a] | 68.9 | 81.4 | Pioglitazone | No use of TZDs | 19 /210 | 357 /6865 | Rate ratio 1.83 (1.10, 3.05) | Excessive alcohol use, obesity, smoking status, HbA1c, previous bladder conditions, previous cancer (other than non-melanoma skin cancer), Charlson comorbidity score, and ever use of other antidiabetic agents.<br>Matched on year of birth, year of cohort entry, sex, and duration of follow-up |
| Chang et al,[8] (2012) | Nested case/control | Taiwan | T2DM | 7.9 (median) | Y[a] | N | 70.9 | 66.8 | Pioglitazone | No use | 84 /401 | 1499 /7490 | OR 0.95 (0.70, 1.29) | Pioglitazone, rosiglitazone, short-acting human insulin, metformin (mean daily dosage in quartiles), sulfonylurea (mean daily dosage in quartiles), number of oral anti-diabetic agents, nephropathy, glinides, ACE inhibitors, chronic kidney disease, calcium channel blockers, |

| | | | | | | | | | | | | | | |
|---|---|---|---|---|---|---|---|---|---|---|---|---|---|---|
| | | | | | | | | | | | | | | neuropathy.<br>Matched on age and sex |
| Mamtani et al,[15] (2012) | Cohort | Europe | T2DM | 3.6 (median) | Y[a] | N | 62.6[d] | 57.2 | Pioglitazone | Rosiglitazone | 41 /10900 | 86 /17614 | HR 1.14 (0.79, 1.66) | Age (<60, 60–69, ≥70 years), sex, smoking (ever vs. never), history of myocardial infarction, and past sulfonylurea use |
| Neumann et al,[6] (2012) | Cohort | France | DM | 3.125 (mean) | Y[a] | Y[a] | 63.2 | 53.4 | Pioglitazone | No use | 175 /155535 | 1841 /1335525 | HR 1.22 (1.05, 1.43) | Age, sex (when applicable) and exposure to glucose-lowering drugs |
| Song et al,[16] (2012) | Case–control | Korea | T2DM | NR | N | N | 69.4 | 84.2 | Pioglitazone | No use | 21 /120 | 308 /867 | OR 2.09 (0.26,16.81) | Smoking<br>Matched on sex and age. |
| Wei et al,[17] (2012) | Cohort | UK | T2DM | 4.4 (mean) | N | N | 64.6 | 54.4 | Pioglitazone | No use | 66 /23548 | 803 /184166 | HR 1.16 (0.83,1.62) | Age, gender, smoking status, BMI and duration of diabetes |
| Fujimoto et al,[18] (2013) | Cohort | Japan | T2DM | NR | Y[a] | N | NR | NR | Pioglitazone | No use | 9 /663 | 673 /20672 | Unadjusted HR 1.75 (0.89,3.45) | NR |
| Vallarino et al,[2] (2013) | Cohort | US | T2DM | 2.1 (mean) | N | N | 58.6 | 57.5 | Pioglitazone | Insulin | 84 /38588 | 44 /17948 | HR 0.92 (0.63,1.33) | Demographics (age, sex, tobacco use), use of medications (defined as any prescription claim within 180 days prior to the index date) and medical history (defined as any diagnosis claim prior to the index date) |
| Hsiao et al,[19] (2013) | Nested case/control | Taiwan | T2DM | 3.68 (mean) | Y[a] | N | 66.3 | 68.5 | Pioglitazone | No use | 153 /676 | 3259 /19796 | OR 1.62 (0.92,2.86) | Duration of diabetes, chronic renal failure, bladder conditions (calculus of kidney, ureter, lower urinary tract, cystitis and urinary tract infection) and COPD, other hypoglycemic agents, including sulfonylureas, biguanides, a-glucosidase |

| | | | | | | | | | | | | | | |
|---|---|---|---|---|---|---|---|---|---|---|---|---|---|---|
| | | | | | | | | | | | | | | inhibitors and insulin. Matched on sex and age. |
| Balaji et al,[20] (2014) | Cohort | India | DM | NR | N | N | NR | NR | Pioglitazone | No use | 1 /31 | 19 /1046 | NR | NR |
| Lee et al,[21] (2014) | Cohort | Taiwan | T2DM | NR | Y[b] | Y[b] | NR | 47.5 | Pioglitazone | No use | 12 /3497 | 72 /31473 | HR 1.03 (0.45,2.35) | Sex, age, duration of diabetes, other diabetes medications, income (monthly income, NT$20,000; monthly income, NT$20,000), residential area, nephritis, chronic kidney disease, kidney infections, hydronephrosis, calculus of the lower urinary tract, cystitis, other disorders of the urethra and urinary tract, hypertension and hyperlipidemia. |
| Jin et al,[22] (2014) | Cohort +Nested case/control | Korea | T2DM | NR | Y[c] | N | 63.4 | 53.3 | Pioglitazone | No use | 30 /11240 | 237 /101953 | HR 1.13 (0.77, 1.68) | Age and sex |
| Kuo et al,[23] (2014) | Nested case/control | Taiwan | DM | NR | N | Y[c] | 69.6 | 61.8 | Pioglitazone | No use | 15 /67 | 244 /1228 | OR 1.20 (0.58, 2.49) | Nephropathy, urinary tract diseases, urinary tract infection, urinary tract stone, hypertension, chronic obstructive pulmonary disease, stroke, ischemic heart disease, peripheral arterial diseases, eye disease, and dyslipidemia. Matched on sex, age, and time from entry into cohort to the index date. |
| Lewis et al,[3] (2015) | Cohort +Nested case/control | US | DM | 7.2 (median) | Y[a] | Y[a] | Reported by segment | 53.5 | Pioglitazone | No use | 186 /34181 | 1075 /158918 | HR 1.06 (0.89, 1.26) | Age, sex, and year of cohort entry, use of other diabetes medication, smoking, race/ethnicity, other diabetes medications, other bladder conditions, median household income, congestive heart |

| | | | | | | | | | | | | | | |
|---|---|---|---|---|---|---|---|---|---|---|---|---|---|---|
| | | | | | | | | | | | | | | failure, cancer other than bladder cancer, renal insufficiency, HbA1c and the interaction with new diagnosis of diabetes, and duration of diabetes, the 3-level time-updated proteinuria testing variable (no testing, negative and positive testing result for proteinuria) |
| Erdmann et al,[5] (2016) | Cohort | Europe | T2DM | 7.8 (mean) | N | N | NR | NR | Pioglitazone | Placebo | 27 /2605 | 26 /2633 | Unadjusted relative risk 1.05 (0.61, 1.79) | NR |
| Tuccori et al,[24] (2016) | Cohort | UK | T2DM | 4.8 (mean) | Y[a] | Y[a] | 63.7 | 56.8 | Pioglitazone | No use of TZDs | 54 /921 | 479 /142758 | HR 1.63 (1.22, 2.19) | Age, year of cohort entry, sex, alcohol related disorders, smoking status, obesity, HbA1c, previous cancer, bladder conditions, Charlson comorbidity score, duration of treated diabetes, and urine protein testing. |
| Korhonen et al,[25] (2016) | Cohort | Europe | T2DM | 2.9 (mean) | Y[c] | Y[c] | 63.2 | 56.3 | Pioglitazone | No use | 130 /56337 | 970 /317109 | HR 1.00 (0.83, 1.21) | age, sex, metformin use, sulfonylurea use, insulin use, use of other diabetes drugs, history of relevant comorbidities, history of other relevant medications, history of bladder comorbidities. |
| Gupta et al,[4] (2015) | Cohort | India | T2DM | NR | N | N | 52.0 | 63.5 | Pioglitazone | No use | 0 /1111 | 0 /1111 | NR | NR |

| **Author (year)** | **Region** | **No. of study sites** | **Study phase** | **Target disease** | **Drug treatments used across groups** | **Intervention group** | | **Control group** | | **Duration of treatment (weeks)** |
|---|---|---|---|---|---|---|---|---|---|---|
| | | | | | | **Type** | **Events/analyzed patients (No.)** | **Type** | **Events/analyzed patients (No.)** | |
| NCT00174993 (2005) | Europe | 321 | III | T2DM | None | Pioglitazone | 14/2605 | Placebo | 6/2633 | 48 months |

| | | | | | | | | | | |
|---|---|---|---|---|---|---|---|---|---|---|
| NCT00225277 (2008) | America | 97 | III | T2DM | None | Pioglitazone | 0/270 | Glimepiride | 1/273 | 72 weeks |
| NCT00494312 (2009) | US | 171 | IV | T2DM | Glyburide placebo-matching capsules or Pioglitazone placebo-matching tablets | Pioglitazone | 2/1051 | Glyburide | 0/1046 | 156 weeks |
| NCT00736099 (2011) | Multi-regions | 232 | III | T2DM | Linagliptine | Pioglitazone | 0/589 | None | 1/1532 | 78 weeks |

Abbreviations: DM, diabetes mellitus; T2DM, type 2 diabetes mellitus; TZDs, thiazolidinediones; 95% CI, 95% confidence interval; HR, hazard ratio; OR, odds ratio; NR, not reported; COPD, chronic obstructive pulmonary disease; HbA1c, hemoglobin A1c concentration; ACE, angiotensin-converting enzyme; BMI, body mass index.

[a]Trials used the same categories that were used in the interim analysis of the Kaiser Permanente Northern California (KPNC) study.

[b]Trials used categories which were partly consistent with the one in the interim analysis of the KPNC study.

[c]Trials used categories which were inconsistent with the one in the interim analysis of the KPNC study.

[d]Median age (years).

**eTable 2**. Quality Assessment of Studies Regarding Bladder Cancer Risk and Pioglitazone Use

| **Author (year)** | **Ascertainment of diabetes** | **Representativeness of the exposed cohort** | **Selection of the non-exposed cohort** | **Ascertainment of exposure to pioglitazone** | **Demonstration that outcome of interest was not present at start of study** | **Comparability of study controls for important factors** | **Assessment of outcome** | **Completeness of outcome** | **Score** |
|---|---|---|---|---|---|---|---|---|---|
| Tseng et al,[13] (2012) | T2DM were identified based on ICD-9 codes 250.1–250.9 | Generally representative of the population in corresponding region | Drawn from the same population as the exposed cohort | Statement not explicit; likely from the drug prescription in the electronic medical records | Yes, patients who had bladder cancer before entry were excluded | Cox regression was used to estimate confounders, including age, sex, diabetes duration, nephropathy, urinary tract disease, hypertension, other combined disease, other diabetes medications, statin, fibrate, ACE inhibitor/angiotensin receptor blocker, calcium channel blocker, region of residence, occupation, and other cancer before baseline, extracted by ICD-9 codes | Bladder cancer was identified based on ICD-9 codes 188.xx | Authors did not mention the completeness of outcome | 8 |
| Mamtani et al,[15] (2012) | Statement not explicit; T2DM likely identified from database | Generally representative of the population in corresponding region | Drawn from the same population as the exposed cohort | Drug prescription in the electronic medical records | Yes, patients who had bladder cancer before entry were excluded | Cox proportional hazards regression models were used to estimate potential confounders, including other diabetes medications and variables believed to affect either the risk of bladder cancer or the probability of receiving a TZD | Bladder cancer was identified by The Read Codes (Version 2) | Authors did not mention the completeness of outcome | 8 |

| | | | | | | | | | |
|---|---|---|---|---|---|---|---|---|---|
| Neumann et al,[6] (2012) | Statement not explicit; DM likely from identifying ICD-9 code in the database | Generally representative of the population in corresponding region | Drawn from the same population as the exposed cohort | Drug prescription in the electronic medical records | Yes, patients had bladder cancer diagnosed before study entry or within the first 6 months after study entry were excluded | Cox proportional hazard models were used to estimate covariates, including age, sex and use of other glucose-lowering drugs | Bladder cancer was identified by ICD-10 code C67 and specific surgical procedure and/or intravesical instillation of pharmacological product by urethral catheter and/or chemotherapy and/or radiation therapy performed during the same hospital stay | Authors did not mention the completeness of outcome | 8 |
| Wei et al,[17] (2012) | T2DM was defined based on the record of General Practice Research Database (GPRD) during the study period | Generally representative of the population in corresponding region | Drawn from the same population as the exposed cohort | Drug prescription in the electronic medical records | Yes, the study outcome was incident bladder cancer during the follow-up period | Cox proportional hazard model was used to adjust for age, gender, smoking status, BMI and duration of diabetes | Data collected during the follow up | There were 45 and 3683 subjects with missing data about smoking history, and 499 and 11948 subjects with missing data about BMI in pioglitazone treatment group and other oral hypoglycaemic drugs treatment group, respectively | 9 |

| | | | | | | | | | |
|---|---|---|---|---|---|---|---|---|---|
| Fujimoto et al,[18] (2013) | Statement not explicit; T2DM identified likely from database | NR | Selection of the non-exposed cohort | Statement not explicit; likely from the drug prescription in the database | Yes, patients who had bladder cancer before entry were excluded. | No adjusted analysis was conducted | Statement not explicit; likely identified from the database | Authors did not mention the completeness of outcome | 5 |
| Vallarino et al,[2] (2013) | T2DM identified by ICD-9 codes 250.x0 or 250.x2 | Generally representative of the population in corresponding region | Drawn from the same population as the exposed cohort | Statement not explicit; likely from the drug prescription in the database | Yes, patients who had bladder cancer before entry were excluded | Cox regression models were used to estimate covariates, including demographics, use of medications and medical history | Bladder cancer was identified based on ICD-9 codes 188.xx, 233.7 | Authors did not mention the completeness of outcome | 8 |
| Balaji et al,[20] (2014) | DM was identified by medical records screening from the hospital | NR | Drawn from the same population as the exposed cohort | Statement not explicit; likely identified by medical records screening from the hospital | NR | NR | Bladder cancer cases were identified by medical records screening from the hospital | Authors did not mention the completeness of outcome | 4 |
| Lee et al,[21] (2014) | T2DM identified by ICD-9-CM code 250.1–250.9 | Generally representative of the population in corresponding region | Drawn from the same population as the exposed cohort | From the drug prescription in the database | Yes, patients who had bladder cancer before entry were excluded. | Cox regression analysis was used to estimate confounders, including sex, age, duration of diabetes, other diabetes medications, income, residential area, nephritis, chronic kidney disease, kidney infections, hydronephrosis, calculus of the lower urinary tract, cystitis, other disorders of the urethra and urinary tract, hypertension and hyperlipidemia | Bladder cancer cases were identified according to ICD-9-CM code 188 and were confirmed by the issuance of catastrophic illness cards | Authors did not mention the completeness of outcome | 8 |

| | | | | | | | | | |
|---|---|---|---|---|---|---|---|---|---|
| Jin et al,[22] (2014) | Statement not explicit; T2DM likely identified from database | NR | Drawn from the same population as the exposed cohort | From the drug prescription in the database | Yes, patients who had bladder cancer before entry were excluded | Cox regression model was used to control for age at baseline and sex | Bladder cancer cases were identified according to ICD-10 code C67 | Authors did not mention the completeness of outcome | 6 |
| Lewis et al,[3] (2015) | DM identified basis of hospital and physician diagnoses, prescription medications, and laboratory tests. | Generally representative of the population in corresponding region | Drawn from the same population as the exposed cohort | From the drug prescription in the database | Yes, patients with a diagnosis of bladder cancer before entry or within 6 months of entry were excluded | Cox regression analysis was used to estimate confounders, including age, sex, and year of cohort entry, use of other diabetes medication, smoking, race/ethnicity, other diabetes medications, other bladder conditions, median household income, congestive heart failure, cancer other than bladder cancer, renal insufficiency, HbA1c and the interaction with new diagnosis of diabetes, and duration of diabetes, the 3-level time-updated proteinuria testing variable | Bladder cancer cases were identified from the KPNC cancer registry, which reports to the California Cancer Registry and the National Cancer Institute's Surveillance, Epidemiology and End Results program of registries | There were missing data regarding race/ethnicity, renal function at baseline, income, baseline HbA1c, and diabetes duration at baseline, in the pioglitazone group and never use group, respectively | 9 |
| Erdmann et al,[5] (2016) | T2DM patients identified by physicians | NR | Drawn from the same population as the exposed cohort | Drug prescription in the routine clinical practice | Statement not explicit; 11 patients were diagnosed bladder cancer within 1 years | There were no adjustments have been done. | Statement not explicit; likely from the data collected during the follow up | A similar percentage of patients on pioglitazone or placebo groups withdrew consent to follow-up (<1%), were confirmed lost to follow-up | 6 |

| | | | | | | | | | |
|---|---|---|---|---|---|---|---|---|---|
| | | | | | | | | (~9%), and had an unconfirmed status [i.e. last contact ≥9 years (<5%)]. | |
| Tuccori et al,[24] (2016) | T2DM was identified by Read code from the database | Generally representative of the population in corresponding region | Drawn from the same population as the exposed cohort | A coded drug dictionary based on the UK Prescription Pricing Authority Dictionary is used to record prescriptions | Yes, patients who had bladder cancer before entry were excluded | Time dependent Cox proportional hazards models were used to estimate covariates, including age, sex, year of cohort entry, body mass index, smoking status, alcohol related disorders, HbA1cace, duration of treated diabetes, previous bladder conditions, history of cancer, presence of at least one urine protein test in the year before cohort entry, and Charlson comorbidity score 26 | Bladder cancer was identified by Read code from the database | There were 9 and 1530 patients with unknown data about BMI, 14 and 3434 patients with unknown data about smoking, 236 and 49,480 patients with unknown data about HbA1c, in pioglitazone group and no pioglitazone group, respectively | 9 |
| Korhonen et al,[25] (2016) | T2DM was identified from database | Generally representative of the population in corresponding region | Drawn from the same population as the exposed cohort | Drug use data were based on outpatient prescription data and outpatient dispensing data | Yes, patients with histories of malignant or benign bladder neoplasms were excluded | Age, sex, metformin use, sulfonylurea use, insulin use, use of other diabetes drugs, all exact matching variables, propensity scores, and all propensity score variables evaluated at cohort entry date | Bladder cancer cases were identified from cancer registries and hospital records | Authors did not mention the completeness of outcome | 8 |

| | | | | | | | | | | |
|---|---|---|---|---|---|---|---|---|---|---|
| Gupta et al,[4] (2015) | Statement not explicit; T2DM likely identified from database | NR | Drawn from the same population as the exposed cohort | Statement not explicit; likely from the drug prescription in the database | NR | NR | | Bladder cancer was assessed by taking history and detailed urinary analysis including urine routine, microscopy, hematuria, and cytology examination | Authors did not mention the completeness of outcome | 4 |

| **Author (year)** | **Is case definition adequate** | **Ascertainment of diabetes** | **Representativeness of the cases** | **Selection of controls** | **Definition of controls** | **Comparability of study controls for important factors** | **Ascertainment of exposure to pioglitazone** | **Same method of ascertainment for exposure to pioglitazone in both arms** | **Completeness of data within database** | **Score** |
|---|---|---|---|---|---|---|---|---|---|---|

| | | | | | | | | | | |
|---|---|---|---|---|---|---|---|---|---|---|
| Azoulay et al,[14] (2012) | Statement not explicit; likely from identifying the clinical diagnoses according to the ICD codes | Statement not explicit; T2DM likely from identifying patients newly treated with noninsulin antidiabetic drugs | Representative of the population in corresponding region | Up to 20 controls were randomly selected from the case's risk set, after matching on year of birth, year of cohort entry, sex, and duration of follow-up. And all controls were alive, had no previous diagnosis of bladder cancer | Patients had no previous diagnosis of bladder cancer | Conditional logistic models were used to control for year of birth, year of cohort entry, sex, and duration of follow-up,HbA1c, excessive alcohol use, obesity, smoking, previous cancer, previous bladder conditions, and Charlson comorbidity score | Statement not explicit; likely from the drug prescription in the electronic medical records | Yes, both cases and controls who had been prescribed TZDs identified with claims database | Authors did not mention the completeness of data in the database, however, HbA1c information was reported as missing for 19% of the cases and controls | 9 |
| Chang et al,[8] (2012) | All potential cases were validated by a linkage through National Cancer Registry | T2DM patients were identified by diagnostic codes (ICD-9-CM) | Obviously representative series of cases in corresponding region | A risk-set sampling matched by age, sex, and the number of days of follow-up was used to find controls for the cohort | Patients had no previous diagnosis of bladder cancer | Conditional logistic regression was used to test potential covariates, including socioeconomic status, diabetes complications and comorbidities at cancer diagnosis, other antidiabetic agents, antihypertensive medications, statin, and aspirin | From the outpatient pharmacy prescription database | Statement not explicit; likely from the outpatient pharmacy prescription database | Authors did not mention the completeness of data in the database | 9 |

| | | | | | | | | | | |
|---|---|---|---|---|---|---|---|---|---|---|
| Song et al,[16] (2012) | Bladder cancer diagnosis confirmed by cytology | Statement not explicit; T2DM patients likely identified from electronic medical records | Obviously representative series of cases in corresponding region | Age- and sex-matched diabetics without bladder cancer were enrolled as the control group | Patients had no previous diagnosis of bladder cancer | Multivariate conditional logistic regression model was used to determine the factors, such as, age, sex, duration of diabetes, obesity, alcohol, smoking, and anti-diabetic agents, and potential underlining medical conditions | Statement not explicit; likely from the drug prescription in the electronic medical records | Statement not explicit; likely from the drug prescription in the electronic medical records | Authors did not mention the completeness of data in the database, but to avoid incomplete or missing data on potential confounders, authors were selected from the Severance diabetes registry | 9 |
| Hsiao et al,[19] (2013) | Diagnosis of bladder cancer was identified by ICD-9-CM codes: 188.xx | Diagnosis of T2DM identified by ICD-9-CM code 250.xx | Obviously representative series of cases in corresponding region | For each case, five matched controls were randomly selected from the same diabetic cohort using the incidence density sampling approach, and were matched to the cases for age, sex and entry date | Statement not explicit; likely patients without bladder cancer | Multivariable conditional logistic regressions were used to estimate the associated factors, including duration of diabetes, co-morbid conditions and concomitant medications. Co-morbid conditions included chronic renal failure, bladder conditions and chronic obstructive pulmonary disease (COPD) | The exposure to pioglitazone was based on identified prescriptions | Yes, both cases and controls who had been prescribed pioglitazone identified from claims database | Authors did not mention the completeness of data in the database | 9 |

| | | | | | | | | | | |
|---|---|---|---|---|---|---|---|---|---|---|
| Kuo et al,[23] (2014) | Diagnoses of bladder cancer was identified by ICD-9 188.** codes | T2DM patients were identified by diagnostic codes for diabetes ICD-9-CM | Obviously representative series of cases in corresponding region | For each case, four control individuals were randomly selected from the set of all eligible controls, and matched to the cases by sex, age, and time from entry into cohort to the index date | Patients had no previous diagnosis of bladder cancer | Conditional logistic regression model was used to estimate relative factors, including documented risk factors for bladder cancer and comorbidities, were retrieved by ICD-9 codes | Information on pioglitazone exposure was extracted from the prescription database | Statement not explicit, likely extracted from the prescription database | Authors did not mention the completeness of data in the databaseBMIH | 9 |

| Author (year) | Randomization sequence generation | Allocation concealment | Blinding of participants and personnel | Blinded assessment bladder cancer events | Incomplete outcome data | Selective reporting |
|---|---|---|---|---|---|---|
| NCT00174993 (2005) | Low | Low | Low | Low | Low[a] | Low |
| NCT00225277 (2008) | Low | Low | Low | Low | Low[a] | Low |
| NCT00494312 (2009) | Low | Low | Low | Low | Low[a] | Low |
| NCT00736099 (2011) | Unclear | Unclear | Unclear | Unclear | NR[a] | Unclear |

Abbreviations: DM, diabetes mellitus; T2DM, type 2 diabetes mellitus; ICD, international Classification of diseases; ACE, angiotensin-converting enzyme; TZDs, thiazolidinediones; NR, not reported; HbA1c, hemoglobin A1c concentration; BMI, body mass index

[a] Data extracted from serious adverse events.

**eReferences.** For eFigure 1, 2, 5, 8 and 9, eTable 1 and 2


1. Ferrara A, Lewis JD, Quesenberry CP, Jr., Peng T, Strom BL, Van Den Eeden SK, Ehrlich SF, Habel LA: **Cohort study of pioglitazone and cancer incidence in patients with diabetes**. *Diabetes Care* 2011, **34**(4):923-929.
2. Vallarino C, Perez A, Fusco G, Liang H, Bron M, Manne S, Joseph G, Yu S: **Comparing pioglitazone to insulin with respect to cancer, cardiovascular and bone fracture endpoints, using propensity score weights**. *Clin Drug Investig* 2013, **33**(9):621-631.
3. Lewis JD, Habel LA, Quesenberry CP, Strom BL, Peng T, Hedderson MM, Ehrlich SF, Mamtani R, Bilker W, Vaughn DJ *et al*: **Pioglitazone Use and Risk of Bladder Cancer and Other Common Cancers in Persons With Diabetes**. *JAMA* 2015, **314**(3):265-277.
4. Gupta S, Gupta K, Ravi R, Mehta V, Banerjee S, Joshi S, Saboo B: **Pioglitazone and the risk of bladder cancer: An Indian retrospective cohort study**. *Indian J Endocrinol Metab* 2015, **19**(5):639-643.
5. Erdmann E, Harding S, Lam H, Perez A: **Ten-year observational follow-up of PROactive: a randomized cardiovascular outcomes trial evaluating pioglitazone in type 2 diabetes**. *Diabetes Obes Metab* 2016, **18**(3):266-273.
6. Neumann A, Weill A, Ricordeau P, Fagot JP, Alla F, Allemand H: **Pioglitazone and risk of bladder cancer among diabetic patients in France: a population-based cohort study**. *Diabetologia* 2012, **55**(7):1953-1962.
7. Tseng CH: **Pioglitazone does not affect the risk of kidney cancer in patients with type 2 diabetes**. *Metabolism* 2014, **63**(8):1049-1055.
8. Chang CH, Lin JW, Wu LC, Lai MS, Chuang LM, Chan KA: **Association of thiazolidinediones with liver cancer and colorectal cancer in type 2 diabetes mellitus**. *Hepatology* 2012, **55**(5):1462-1472.
9. Tseng CH: **Pioglitazone does not affect the risk of ovarian cancer: analysis of a nationwide reimbursement database in Taiwan**. *Gynecol Oncol* 2013, **131**(1):135-139.
10. Tseng CH: **Pioglitazone and oral cancer risk in patients with type 2 diabetes**. *Oral Oncol* 2014, **50**(2):98-103.
11. Chang SS, Hu HY: **Association of thiazolidinediones with gastric cancer in type 2 diabetes mellitus: a population-based case-control study**. *BMC Cancer* 2013, **13**:420.
12. Tseng CH: **Pioglitazone and thyroid cancer risk in Taiwanese patients with type 2 diabetes 2**. *J Diabetes* 2014, **6**(5):448-450.
13. Tseng CH: **Pioglitazone and bladder cancer: a population-based study of Taiwanese**. *Diabetes Care* 2012, **35**(2):278-280.
14. Azoulay L, Yin H, Filion KB, Assayag J, Majdan A, Pollak MN, Suissa S: **The use of pioglitazone and the risk of bladder cancer in people with type 2 diabetes: nested case-control study**. *BMJ* 2012, **344**:e3645.
15. Mamtani R, Haynes K, Bilker WB, Vaughn DJ, Strom BL, Glanz K, Lewis JD: **Association between longer therapy with thiazolidinediones and risk of bladder cancer: a cohort study**. *J Natl Cancer Inst* 2012, **104**(18):1411-1421.
16. Song SO, Kim KJ, Lee BW, Kang ES, Cha BS, Lee HC: **The risk of bladder cancer in korean diabetic subjects treated with pioglitazone**. *Diabetes Metab J* 2012, **36**(5):371-378.
17. Wei L, MacDonald TM, Mackenzie IS: **Pioglitazone and bladder cancer: a propensity score

**matched cohort study**. *Br J Clin Pharmacol* 2012, **75**(1):254-259.

18. Fujimoto K, Hamamoto Y, Honjo S, Kawasaki Y, Mori K, Tatsuoka H, Matsuoka A, Wada Y, Ikeda H, Fujikawa J *et al*: **Possible link of pioglitazone with bladder cancer in Japanese patients with type 2 diabetes**. *Diabetes Res Clin Pract* 2013, **99**(2):e21-23.
19. Hsiao FY, Hsieh PH, Huang WF, Tsai YW, Gau CS: **Risk of bladder cancer in diabetic patients treated with rosiglitazone or pioglitazone: a nested case-control study**. *Drug Saf* 2013, **36**(8):643-649.
20. Balaji V, Seshiah V, Ashtalakshmi G, Ramanan SG, Janarthinakani M: **A retrospective study on finding correlation of pioglitazone and incidences of bladder cancer in the Indian population**. *Indian J Endocrinol Metab* 2014, **18**(3):425-427.
21. Lee MY, Hsiao PJ, Yang YH, Lin KD, Shin SJ: **The association of pioglitazone and urinary tract disease in type 2 diabetic Taiwanese: bladder cancer and chronic kidney disease**. *PLoS One* 2014, **9**(1):e85479.
22. Jin SM SS, Jung CH: **Risk of bladder cancer among patients with diabetes treated with a 15 mg pioglitazone dose in Korea: a multi-center retrospective cohort study**. *J Korean Med Sci* 2014, **29**:5.
23. Kuo HW TM, Ho SC, Yang CY: **Pioglitazone use and the risk of bladder cancer.** *Kaohsiung J Med Sci* 2014, **30**:4.
24. Tuccori M, Filion KB, Yin H, Yu OH, Platt RW, Azoulay L: **Pioglitazone use and risk of bladder cancer: population based cohort study**. *BMJ* 2016, **352**:i1541.
25. Korhonen P, Heintjes EM, Williams R, Hoti F, Christopher S, Majak M, Koolhouweling L, Strongman H, Linder M, Dolin P: **Pioglitazone use and risk of bladder cancer in patients with type 2 diabetes: retrospective cohort study using datasets from four European countries**. *Bmj* 2016, **354**.

**eAppendix 2.** MOOSE Guidelines Checklist

| Criteria | | Brief description of how the criteria were handled in the meta-analysis |
|---|---|---|
| **Reporting of background should include** | | |
| √ | Problem definition | The association between pioglitazone use and bladder cancer risk was announced and debated for many years. |
| √ | Hypothesis statement | Pioglitazone use may be associated with increased bladder cancer risk. |
| √ | Description of study outcome(s) | Bladder and other cancer risks. |
| √ | Type of exposure or intervention used | Pioglitazone use. |
| √ | Type of study designs used | We included observational studies and randomized controlled trials. |
| √ | Study population | Patients with diabetes. |
| **Reporting of search strategy should include** | | |
| √ | Qualifications of searchers | The credentials of all investigators are indicated in the author list. |
| √ | Search strategy, including time period included in the synthesis and keywords | Three authors performed the literature search from the inception through Jan, 5 2017 without language restriction. |
| √ | Databases and registries searched | Embase, PubMed, Web of Science, Cochrane Library, and ClinicalTrials.gov. |
| √ | Search software used, name and version, including special features used | No specific search software was employed. Endnote was used to eliminate duplications. |
| √ | Use of hand searching | We hand-searched bibliographies of retrieved manuscripts for additional references. |
| √ | List of citations located and those excluded, including justification | Details of the literature search process are presented in the Figure 1. |
| √ | Method of addressing articles published in languages other than English | We placed no restrictions on language. If necessary, we resorted to our colleagues who fluent in the corresponding language for translation. |
| √ | Method of handling abstracts and unpublished studies | We contacted a few authors to make clarifications regarding their unpublished studies, but none of these studies were included in our analyses. |
| √ | Description of any contact with authors | Not available. |
| **Reporting of methods should include** | | |
| √ | Description of relevance or appropriateness of studies assembled for assessing the hypothesis to be tested | The inclusion and exclusion criteria were described in the methods section. |
| √ | Rationale for the selection and coding of data | Data extracted from each study were relevant to the study design, population characteristics, exposure and control conditions, outcome, etc. |
| √ | Assessment of confounding | Subgroup and sensitivity analyses were performed to address potential confounders. |

| | | |
|---|---|---|
| √ | Assessment of study quality, including blinding of quality assessors; stratification or regression on possible predictors of study results | The Newcastle-Ottawa quality assessment scale and the Cochrane Collaboration's tool were used to assess the study quality. |
| √ | Assessment of heterogeneity | The heterogeneity was evaluated by the use of I-squared for all analyses. |
| √ | Description of statistical methods in sufficient detail to be replicated | Description of methods, subgroup analyses, sensitivity analyses, and assessment of publication bias are all detailed in the methods section. |
| √ | Provision of appropriate tables and graphics | Five figures, one table, one eAppendixes, nine efigures and two etables were provided. |
| **Reporting of results should include** | | |
| √ | Graphic summarizing individual study estimates and overall estimate | Figure 2 |
| √ | Table giving descriptive information for each study included | eTable 1 |
| √ | Results of sensitivity testing | Results section. |
| √ | Indication of statistical uncertainty of findings | 95% confidence intervals were presented with all summary estimates. |
| **Reporting of discussion should include** | | |
| √ | Quantitative assessment of bias | No significant publication bias presented in our study, and sensitivity analyses were performed to quantify potential biases. |
| √ | Justification for exclusion | We excluded studies that didn't relevant to pioglitazone and cancer incidence. |
| √ | Assessment of quality of included studies | The study quality was reported both in the eTable 2 and sensitivity analysis section. |
| **Reporting of conclusions should include** | | |
| √ | Consideration of alternative explanations for observed results | Alternative explanations were provided in the discussion section. |
| √ | Generalization of the conclusions | Our results are generalizable as we included studies from different population regions. |
| √ | Guidelines for future research | We make recommendations in the discussion section. |
| √ | Disclosure of funding source | This study was supported by grants from National Key R&D Program of China (No. 2016YFC1101100), National Natural Science Foundation of China (No. 81471039, No. 81270893, No. 81228023 and No. 81401601, and No.81402202) and the Natural Science Foundation Project of Chongqing (CSTC2014jcyjjq10006, CSTC2012jjB10023, and CSTC2016jcyjA0518). |
